# The mRNA-binding protein HLN1 enhances drought stress tolerance by stabilizing the *GAD2* mRNA in Arabidopsis

**DOI:** 10.1007/s44154-025-00239-4

**Published:** 2025-06-06

**Authors:** Chuangfeng Liu, Yang Wang, Jialin Peng, Zhengyu Shao, Yajie Liu, Zhiqing Zhang, Xiaoyu Mo, Yilin Yang, Tao Qin, Yiji Xia, Liming Xiong

**Affiliations:** 1 Department of Biology, Hong Kong Baptist University, Kowloon Tong, Hong Kong, China; 2 College of Forestry, Northwest A&F University, Yangling, 712100 China; 3 College of Grassland Agriculture, Northwest A&F University, Yangling, 712100 China; 4 State Key Laboratory of Agrobiotechnology, The Chinese University of Hong Kong, Sha Tin, Hong Kong, China

**Keywords:** Drought, mRNA-binding protein, Condensate, mRNA stability, HLN1, GABA

## Abstract

Drought is a common environmental condition that significantly impairs plant growth. In response to drought, plants close their stomata to minimize transpiration and meanwhile activate many stress-responsive genes to mitigate damage. These stress-related mRNA transcripts require the assistance of RNA-binding proteins throughout their metabolic process, culminating in protein synthesis in the cytoplasm. In this study, we identified HLN1 (Hyaluronan 1), an RNA-binding protein with similarity to the animal hyaluronan-binding protein 4 / serpin mRNA binding protein 1 (HABP4/SERBP1), as crucial for plant drought tolerance. The *hln1* loss-of-function mutant exhibited higher transpiration rates due to impaired stomatal closure, making it highly susceptible to drought. Drought stress increased *HLN1* expression, and the protein underwent liquid–liquid phase separation (LLPS) to form mRNA-ribonucleoprotein (mRNP) condensates in the cytoplasm under osmotic stress. We identified *GAD2* as a potential mRNA target of HLN1. *GAD2* encodes the predominant glutamate decarboxylase synthesizing γ‐aminobutyric acid (GABA), a non-proteinogenic amino acid that modulates stomatal movement. RIP-qPCR and EMSA showed that HLN1 binds *GAD2* mRNA, which promotes HLN1 condensate formation. In *hln1* mutants, *GAD2* transcripts were less stable, reducing steady-state mRNA levels. As a result, *hln1* accumulated less GABA and exhibited impaired stomatal closure under drought. Conversely, *HLN1* overexpression stabilized *GAD2* mRNA, increased GABA levels, and enhanced drought tolerance in transgenic plants. *GAD2* overexpression in *hln1* mutants also rescued the drought-sensitive phenotypes. Overall, our study reveals a mechanism whereby HLN1 stabilizes *GAD2* mRNA to enhance GABA production and drought tolerance. These findings provide novel strategies for engineering drought-resistant crops.

**Supplementary Information:**

The online version contains supplementary material available at 10.1007/s44154-025-00239-4.

## Introduction

Drought stress significantly reduces plant growth and crop productivity. To cope with drought, plants have evolved diverse adaptive responses, such as developing deep root systems for better water foraging, closing stomata to reduce transpiration, and activating thousands of stress-responsive genes whose products mitigate stress damage (Zhu 2016; Gong et al. 2020; Sato et al. 2024). These discrete yet intertwined responses collectively enhance plant drought tolerance. Among these responses, stomatal closure and stress gene expression occur rapidly upon osmotic stress induced by drought and are tightly regulated. In particular, stress gene activation requires coordinated regulation across transcription, processing, and translation, with RNA-binding proteins (RBPs) playing critical roles at each stage. Although thousands of RBPs have been annotated in plant genomes (Ambrosone et al. 2015; Marondedze et al. 2019; Sajeev et al. 2022; Wang et al. 2024), only a limited number have been directly linked to stress responses, and their mechanisms remain poorly understood. Given the importance of RBPs in gene regulation, elucidating their functions in stress responses is vitally important.

The RNA-binding capacity of RBPs is determined by their sequence and structural features. Beyond canonical RNA-binding domains, many RBPs possess intrinsically disordered regions (IDRs) that have the propensity to undergo liquid–liquid phase separation (LLPS) driven by multivalent interactions, particularly under cellular crowding, to form membraneless condensates. These condensates comprise specific RBPs, associated mRNAs and other proteins, yielding distinct granules such as stress granules (SG) and processing bodies (P-bodies), which compartmentalize cellular activities. By concentrating components for transcript processing, storage, modification, translation, or degradation, these dynamic structures facilitate gene regulation (Hentze et al. 2018; Kosmacz et al. 2019; Emenecker et al. 2020; Wiedner and Giudice 2021; Hirose et al. 2023; Wadsworth et al. 2024). While current knowledge about biomolecular condensates is mostly derived from non-plant systems, the prevalence of stress-responsive genes in plants suggests that RBP-driven condensates likely play critical roles in stress signaling and adaptation in plants.

In this study, we identified an RNA binding protein Hyaluronan 1 (HLN1) as an important positive regulator of plant drought tolerance. The *hln1* mutant exhibited elevated transpiration and heightened drought-sensitivity. HLN1 is an RNA-binding protein with similarity to animal hyaluronan-binding protein 4 (HABP4) and serpin mRNA binding protein 1 (SERBP1), and it harbors multiple predicted IDRs. We demonstrated that HLN1 undergoes LLPS to form condensates under osmotic stress. Through computational prediction and experimental analyses, we identified *GAD2* as an HLN1 target. *GAD2* encodes the primary enzyme for the biosynthesis of γ-aminobutyric acid (GABA), a signaling molecule implicating in stomatal regulation (Ramesh et al. 2015; Mekonnen et al. 2016; Xu et al. 2021; Li et al. 2024). We found that HLN1 binds to *GAD2* mRNA, promoting condensate formation and transcript stabilization. Consequently, *GAD2* transcripts degraded more rapidly in *hln1* mutant than in the wild type, leading to reduced GABA levels. Conversely, *HLN1* overexpression enhanced *GAD2* mRNA stability. Consistent with this, *GAD2* overexpression or exogenous GABA supplementation rescued the impaired stomatal closure and drought-sensitive phenotypes of the *hln1* mutant. In summary, our study reveals that HLN1 enhances drought tolerance by stabilizing *GAD2* mRNA and promoting GABA-mediated stomatal regulation under drought stress.

## Results

### HLN1 is required for drought stress tolerance

Upon drought stress, plants reduce stomatal conductance to minimize water loss, leading to elevated leaf temperatures. Using thermal imaging, we isolated an Arabidopsis mutant, *lot1* (*lower temperature 1*), which exhibits higher transpiration rates and greater drought susceptibility (Qin et al. 2019). *LOT1* encodes a protein potentially involved in the posttranslational modifications of stress-signaling proteins, regulating their localization and stability (Qin et al. 2019). To elucidate the modes of action of LOT1, we performed yeast two-hybrid screens (Qin et al. 2019) and conducted co-immunoprecipitation assays with tagged LOT1 transgenic plants. These experiments identified the RNA-binding protein HLN1 (Hyaluronan 1, AT5G47210) as a potential component involved in LOT1-mediated drought responses. We thus investigated further the potential functions of HLN1 in modulating drought stress responses.

**Fig. 1:**
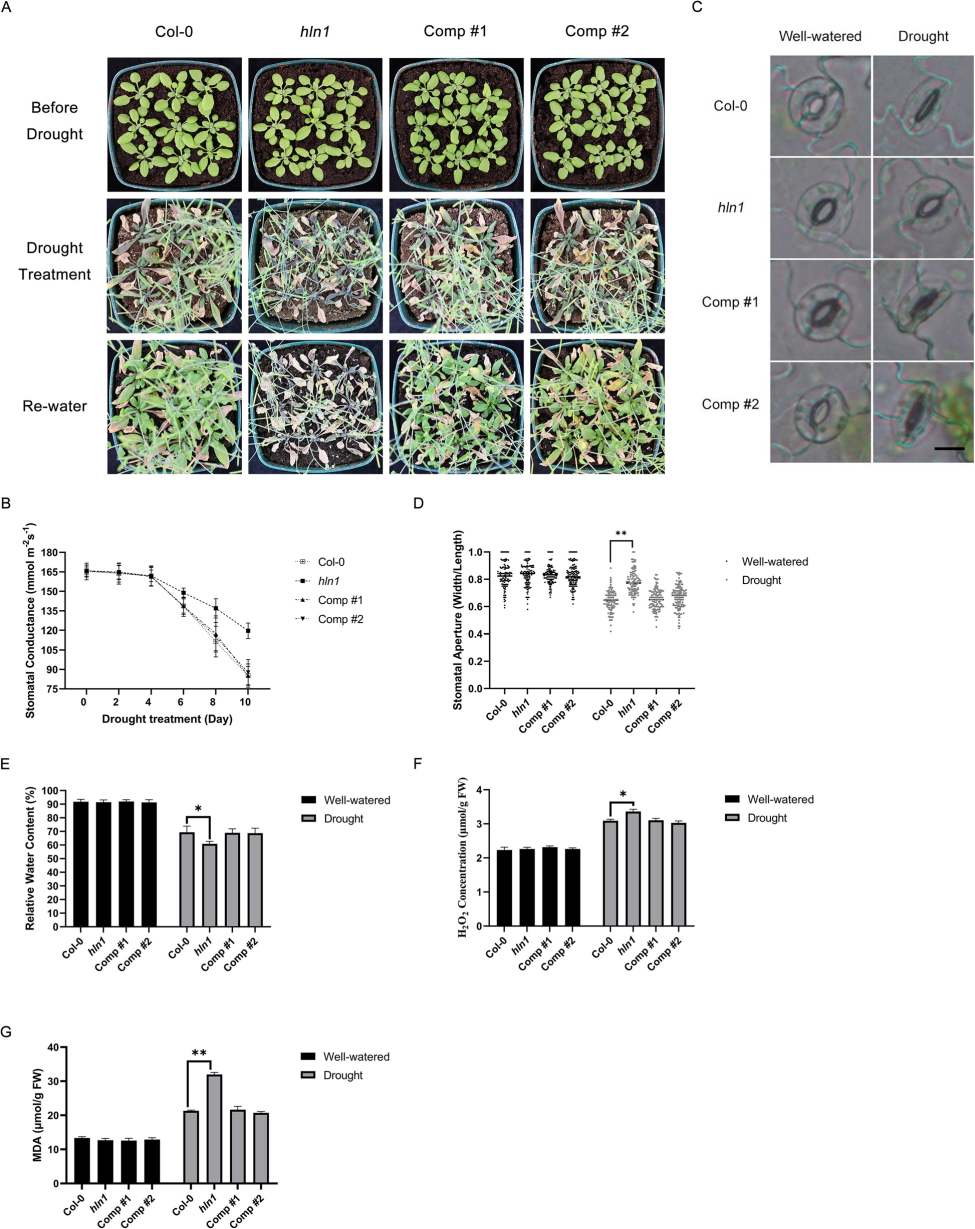
HLN1 is required for drought stress response and tolerance in Arabidopsis. **A** Morphology of Col-0, *hln1*, complementation line #1 and #2 (Comp #1 and #2) plants before (upper panel) and after (middle panel) a 20-day drought stress, and 2 days after rewatering (lower panel). **B** Stomatal conductance in rosette leaves of the indicated plants following drought treatment. Measurements were taken every two days, and data are means ± standard deviations (SD) from three biological replicates. **C** Stomatal morphology of the indicated genotypes under drought treatment. Leaves from 3-week-old plants were excised after 10 days of drought treatment and imaged by light microscope. Experiments were repeated three times with similar results and representative images are shown. Scale bar = 10 μm. **D** Stomatal aperture (expressed as width/length ratio) in leaves of the indicated plants under drought stress. Experiments were repeated three times and stomatal apertures from more than 100 stomata were calculated and shown as dots. Double asterisks (**) represent a *p*-value < 0.01 by Student’s *t*-test. **E** Relative water content (RWC) in rosette leaves of the indicated plants after 10-day drought treatment. Data are means ± SD from three biological replicates. Asterisks (*) indicate a *p*-value < 0.05 by Student’s* t*-test. **F** Hydrogen peroxide (H_2_O_2_) content in drought-treated plants compared to well-watered controls. Data are means ± SD from three biological replicates. Asterisks (*) indicate a *p*-value < 0.05 by Student’s *t*-test. **G** Malondialdehyde (MDA) content in drought-treated plants compared with well-watered plants. Data are means ± SD from three biological replicates. Double asterisks (**) represent a *p*-value < 0.01 by Student’s *t*-test. Plant genotypes: Col-0 (wild type); *hln1* (*hln1* mutant); Comp #1 and #2, two independent complementation lines (i.e., pHLN1-HLN1/*hln1* #1 and #2)

We obtained an *hln1* knockout mutant and two *HLN1* complementation lines (Comp #1 and #2, i.e., pHLN1-HLN1/*hln1* #1 and #2, expressing *HLN1* genomic DNA under its native promoter in the *hln1* mutant background) to assess drought responses. Three-week-old soil-grown plants were subjected to 20 days of water deprivation followed by re-watering. Two days later, the plants were photographed (Fig. 1A). While wild type (Col-0) plants survived, the *hln1* mutant did not. Notably, both complementation lines rescued the *hln1* mutant’s drought sensitivity, confirming that the phenotype resulted from *HLN1* loss (Fig. 1A).

Stomatal conductance of these plants was monitored using a porometer. Significant differences in stomatal conductance between Col-0 and *hln1* mutant became evident four days after water withholding (Fig. 1B). After 10 days of drought, stomatal conductance (mmol m^−2^ s^−1^) decreased to 85.17 (Col-0), 119.60 (*hln1*), 85.23(pHLN1-HLN1/*hln1* #1), and 87.67 (pHLN1-HLN1/*hln1* #2), respectively. The elevated stomatal conductance in *hln1* indicated impaired stomatal regulation. Under the 10-day drought treatment, the stomatal aperture (width-to-length ratio) in Col-0 plants decreased from 0.83 to 0.64 but it only decreased from 0.84 to 0.78 in the *hln1* mutant (Fig. 1C-D). Similar to Col-0, the stomatal aperture in pHLN1-HLN1/*hln1* #1 and #2 plants decreased from 0.84 to 0.66 and from 0.83 to 0.67, respectively (Fig. 1D). These results indicated that the loss of *HLN1* function led to incomplete stomatal closure and excessive transpiration under drought. Consequently, the *hln1* mutant showed significantly lower relative water content (RWC) than Col-0, while complementation lines restored RWC to wild-type levels (Fig. 1E). We also measured stomatal density but found no significant difference between *hln1* and Col-0 (Supplemental Figs. 1A-1B), suggesting that HLN1 does not regulate stomatal development.

Drought stress induces the accumulation of reactive oxygen species (ROS) and malondialdehyde (MDA), a product of membrane lipid peroxidation. We observed significantly higher H_2_O_2_ content in the *hln1* mutant compared to the wild type, while both *hln1* complementation lines showed reduced levels (Fig. 1F). Additionally, MDA levels were significantly elevated in the *hln1* mutant (Fig. 1G), indicating more severe lipid peroxidation. The complementation lines accumulated less MDA than the *hln1* mutant, demonstrating that *HLN1* loss-of-function exacerbates drought-induced cellular damage.

To investigate the effects of *HLN1* overexpression on drought response, we generated transgenic lines expressing *HLN1* under the control of the cauliflower mosaic virus 35S promoter in the Col-0 background. Two independent overexpression lines, both showing over 60-fold higher *HLN1* expression than the wild type (Supplemental Fig. 2), displayed normal growth and development under well-water conditions (Supplemental Fig. 3 A), indicating that *HLN1* overexpression did not impair plant growth. When subjected to 23 days of drought treatment followed by re-watering, *HLN1*-overexpressing plants exhibited enhanced drought tolerance (Supplemental Fig. 3 A). Under the drought treatment, *HLN1*-overexpressing plants showed smaller stomatal aperture (Supplemental Figs. 3D-3E), reduced stomatal conductance, higher RWC, lower H_2_O_2_ accumulation, and decreased MDA levels (Supplemental Figs. 3B-C and H-I). These results demonstrate that overexpressing *HLN1* significantly improves drought tolerance of plants.

**Fig. 2:**
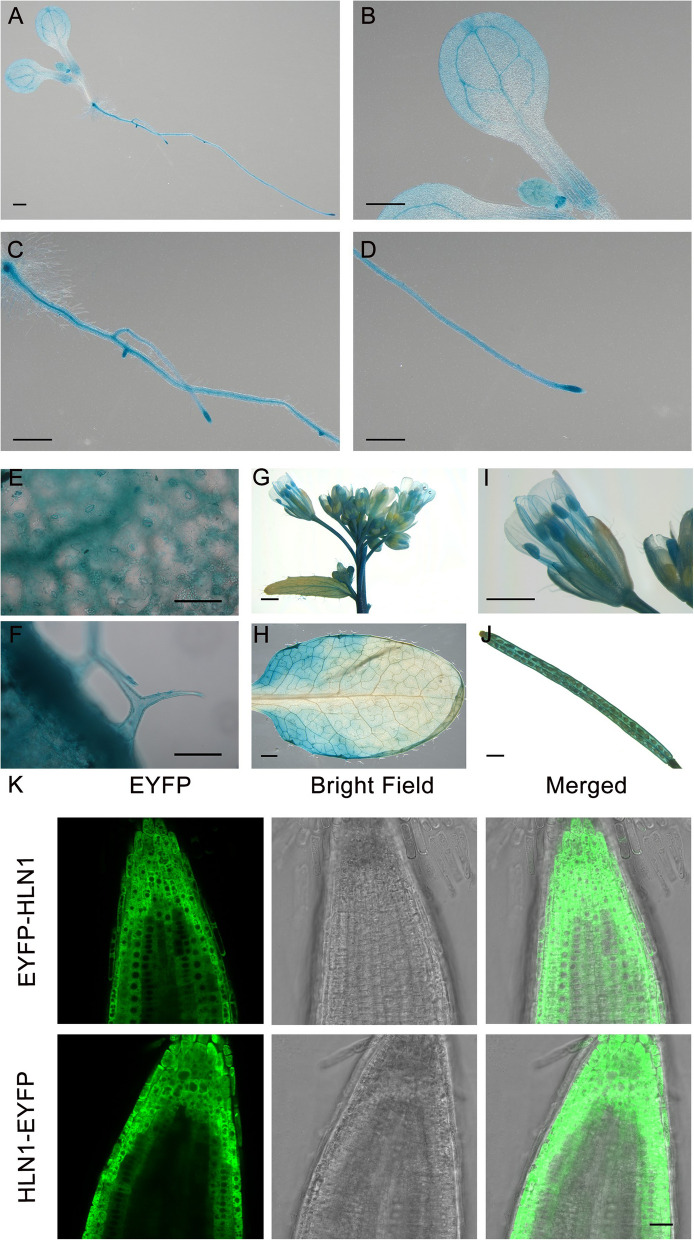
Expression pattern and subcellular localization of HLN1. **A-F** p*HLN1:GUS* expression in 7-day-old seedling (**A**), cotyledons (**B**), lateral roots (**C**), root tip (**D**), guard cells (**E**), and trichomes (**F**). **G-J** p*HLN1:GUS* expression in the inflorescence (**G**), rosette leaf (**H**), flower (**I**), and silique (**J**) of 3-week-old plants. Scale bars: 1000 μm (**A**-**D**, **G**-**J**); 100 μm (**E**–**F**). **K** Subcellular localization of HLN1 in the root tip cells of pGWB 541-HLN1 and pGWB 542-HLN1 transgenic plants. Scale bar in the lower panel (**K**) represents 20 μm

**Fig. 3:**
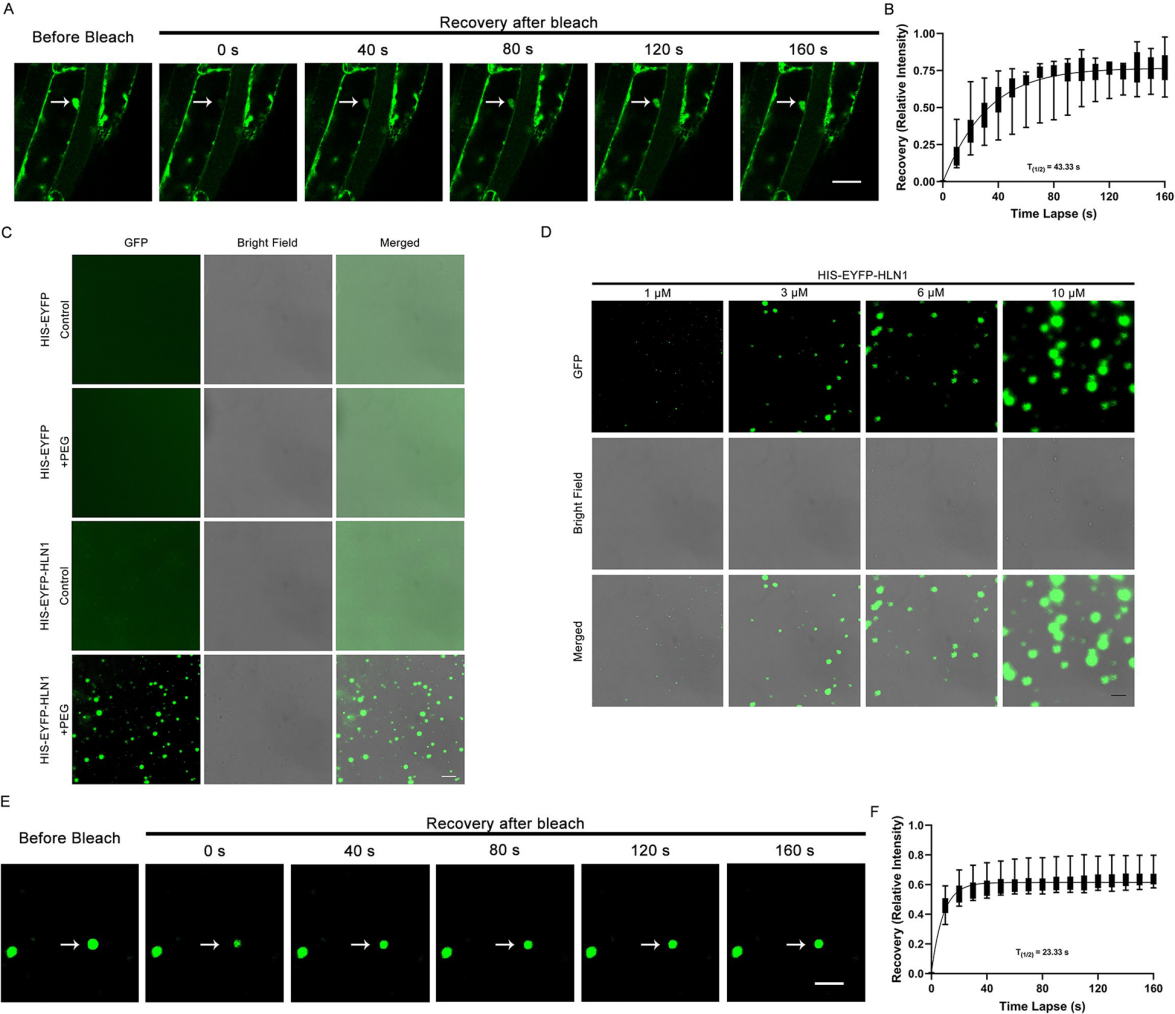
HLN1 forms cytoplasmic condensates via phase-separation both in vivo and in vitro. **A** Fluorescence Recovery After Photobleaching (FRAP) analysis of a HLN1 condensate (arrowhead) in the root elongation zone of an EYFP-HLN1 transgenic seedling. Scale bar = 20 μm. **B** FRAP recovery kinetics of EYFP-HLN1 condensates in transgenic seedlings. The half-time recovery (t_1/2_) was calculated from averaged fluorescence intensity. Error bars represent standard deviations (SD) from 9 biological replicates. **C** in vitro phase separation of HIS-EYFP and HIS-EYFP-HLN1 proteins following the addition of PEG 8000. Scale bar = 20 μm. **D** Concentration-dependent formation of HLN1 condensates with PEG 8000. Scale bar = 20 μm. **E** FRAP analysis of a representative HIS-EYFP-HLN1 droplet. scale bar = 20 μm. **F** FRAP recovery plot of HIS-EYFP-HLN1 droplets. Half-time recovery (t₁/₂) was calculated from averaged intensities. Error bars represent SD from 9 biological replicates

### Expression pattern and subcellular localization of HLN1

To examine the expression pattern of *HLN1,* we generated transgenic Arabidopsis plants expressing the β-*glucuronidase (GUS)* reporter gene driven by a 1,677-bp *HLN1* promoter fragment and performed histochemical staining. In 7-day-old seedlings, GUS signals were detected in cotyledons, lateral roots, root tips, guard cells and trichomes (Fig. 2A-F). In 3-week-old plants, GUS signals were observed in inflorescences, rosette leaves, flowers, and siliques (Fig. 2G-I). Quantitative RT-PCR (qRT-PCR) analysis confirmed higher *HLN1* expression in roots and leaves compared to stems and siliques (Supplemental Fig. 4). Furthermore, *HLN1* expression in 3-week-old plants was significantly enhanced following 10 days of water-withholding (Supplemental Fig. 5).

**Fig. 4:**
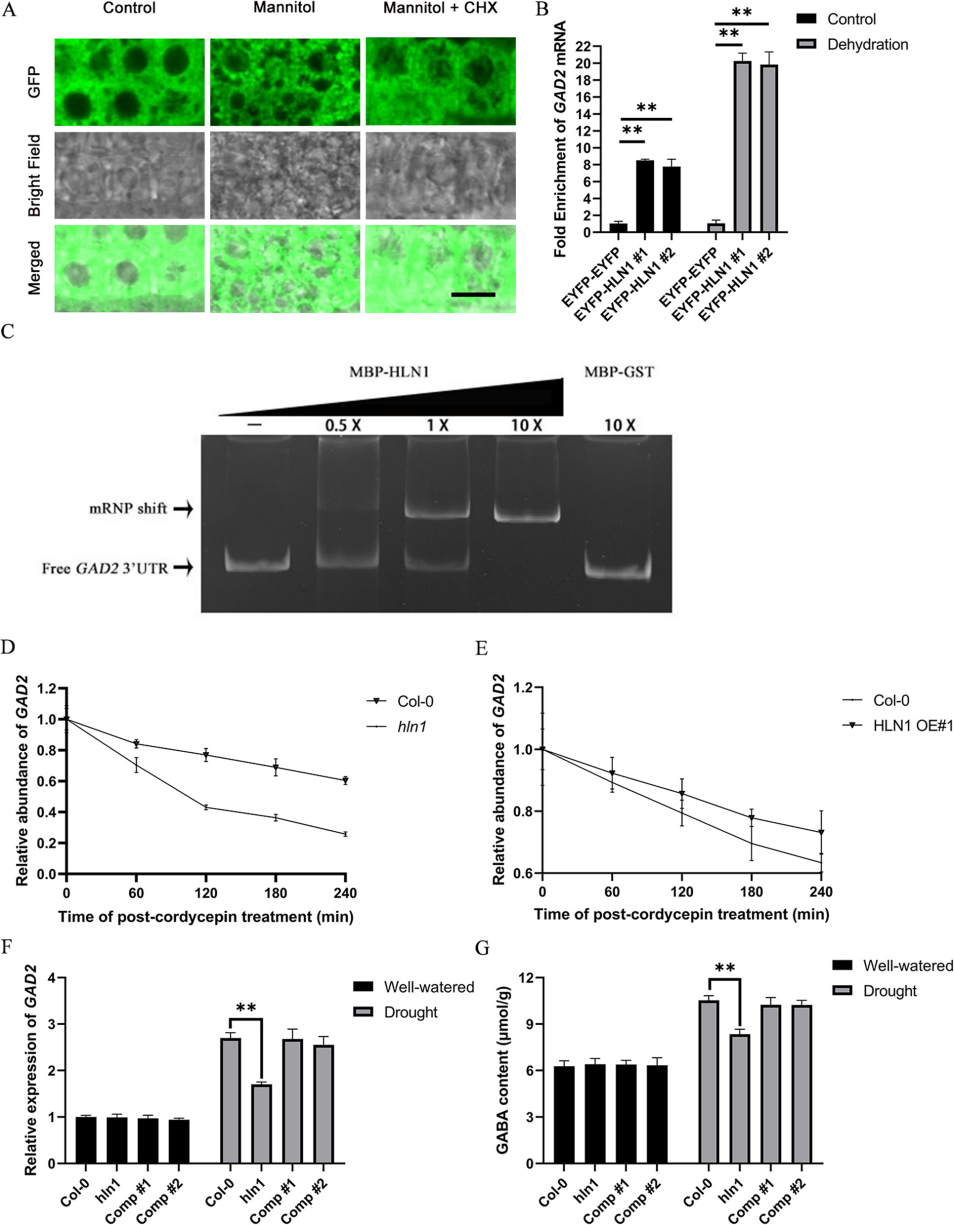
HLN1 interacts with *GAD2* mRNA to regulate its stability in Arabidopsis. **A** Subcellular localization of HLN1 in root tip cells of transgenic lines under normal conditions, mannitol treatment, and mannitol treatment combined with cycloheximide (CHX) treatment. Scale bar = 10 μm. **B** RIP-qPCR analysis of EYFP-HLN1 binding to *GAD2* mRNA with or without dehydration treatment. EYFP-EYFP transgenic line was used as a negative control. Data are means ± SD (*n* = 3 biological replicates). **, *p* < 0.01 by Student’s *t*-test. **C** EMSA of HLN1 binding to *GAD2* 3’UTR analyzed by native PAGE. MBP-GST was negative control. **D** *GAD2* mRNA decay kinetics in *hln1* mutants. Data points show means ± SD (*n* = 3). **E** *GAD2* mRNA decay kinetics in *HLN1* overexpression (OE) lines. Two independent lines showed similar results. Data from one line shown (means ± SD, *n* = 3 per time point). **F** Relative expression of *GAD2* mRNA in Col-0, *hln1,* and two complementation lines (Comp #1 and #2) under drought stress (means ± SD, *n* = 3). **, *p* < 0.01 by Student’s *t*-test. **G** GABA content in Col-0, *hln1* and two complementation lines (Comp #1 and #2) under control or drought stress conditions (mean ± SD, *n* = 3). **, *p* < 0.01 by Student’s *t*-test

**Fig. 5:**
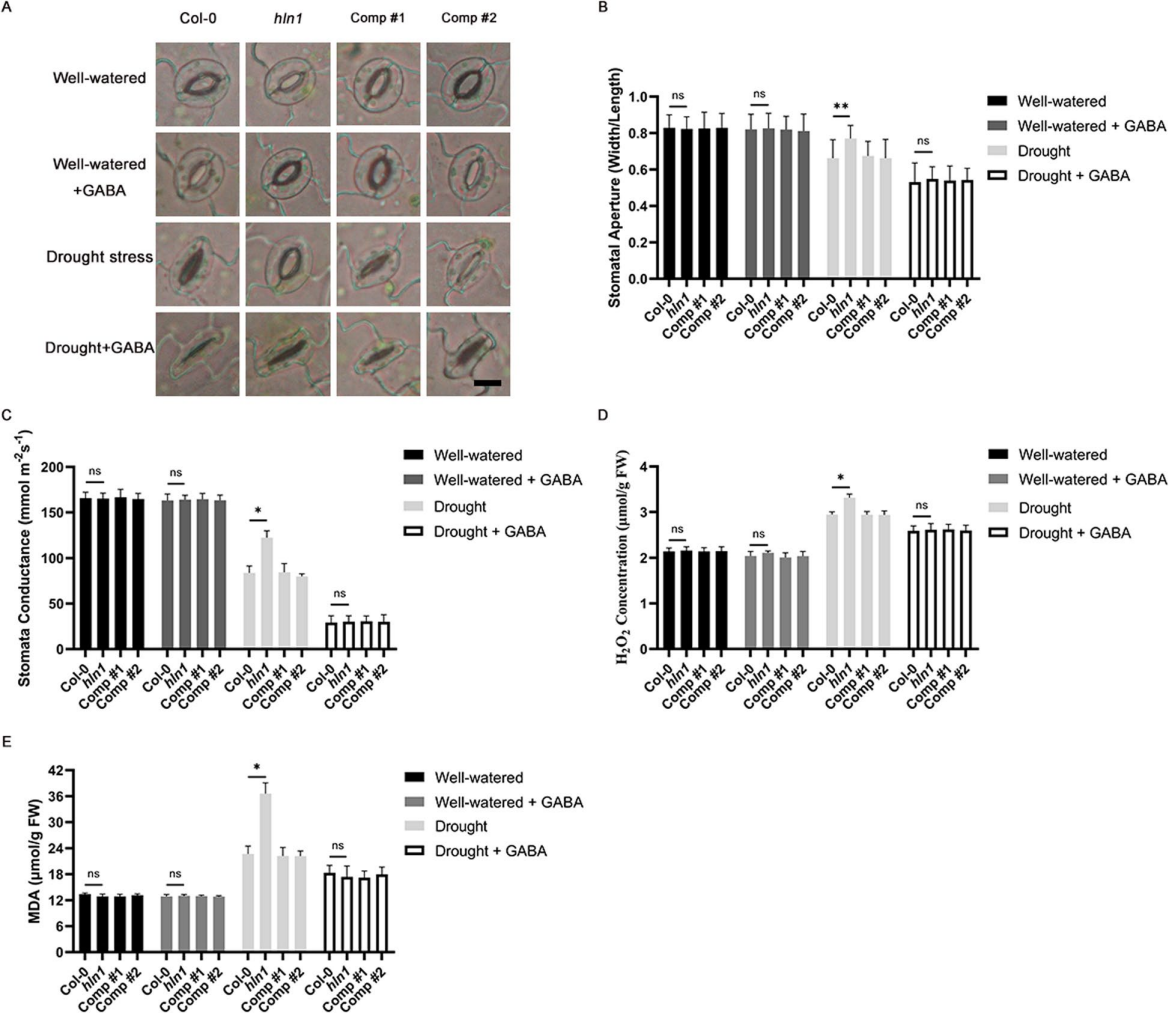
Exogenous GABA treatment reduces stomatal conductance and drought-induced damage in the *hln1* mutant. **A** Stomatal morphology of the indicated genotypes under drought or GABA treatment. Representative results of three independent experiments are shown. Scale bar = 10 μm. **B** Stomatal apertures (expressed as width/length ratio) of the indicated genotypes with or without drought or GABA treatment. Experiments were repeated three times and stomatal apertures from more than 100 stomata were calculated. **C** Stomatal conductance in 3-week-old plants of the indicated genotypes under drought or 4 mM GABA treatment (means ± SD, *n* = 3). **D** Malondialdehyde (MDA) content in 3-week-old plants of the indicated genotypes under drought or 4 mM GABA treatment (means ± SD, *n* = 3). **E** Hydrogen peroxide (H_2_O_2_) content in 3-week-old plants of the indicated genotypes under drought or 4 mM GABA treatment (means ± SD, *n* = 3). Genotypes include Col-0 (wild type); *hln1*(*hln1* mutant); Comp #1 and #2 (two independent *hln1* complementation lines, i.e., pHLN1-HLN1/*hln1* #1 and #2). Error bars represent standard deviation (SD). The double asterisks (**) indicate a *p*-value < 0.01 and the single asterisk (*) indicates a *p*-value < 0.05 by Student’s *t*-test

To determine the subcellular localization of the HLN1 protein, we generated transgenic lines expressing N-terminally-tagged EYFP-HLN1 and C-terminally-tagged HLN1-EYFP under the control of the 35S promoter. Roots of the transgenic lines were examined using a confocal microscope, and HLN1 was found to be localized in the cytoplasm in both EYFP-tagged lines (Fig. 2K).

### HLN1 undergoes liquid–liquid phase separation and forms condensates under osmotic stress

While examining the localization of the EYFP-tagged HLN1 protein, we occasionally observed cytoplasmic condensate-like structures. Structural analysis revealed three potential intrinsically disordered regions (IDRs) in HLN1 (Supplementary Fig. 6), which may drive liquid–liquid phase separation (LLPS) under cellular crowding conditions, leading to membraneless condensate formation. To investigate the phase-separating capability of the HLN1 protein, we treated 7-day-old YFP-HLN1 seedlings with 0.5 M mannitol to induce osmotic stress. While EYFP-HLN1 showed even cytosolic distribution under control conditions, mannitol treatment induced punctuate EYFP-HLN1 aggregates (see below, Fig. 4A). To further determine the nature and properties of these HLN1-containing granules, we incubated 7-day-old EYFP-HLN1 seedlings with cycloheximide (CHX) before the mannitol treatment. It was found that the formation of the HLN1 granules was inhibited by CHX treatment (Fig. 4A), suggesting that these cytoplasmic granules are mRNA-protein (mRNP) condensates similar to stress granules, whose formation is CHX-sensitive (Bounedjah et al. 2014).

We assessed condensate dynamics in the root elongation zone using fluorescence recovery after photobleaching (FRAP). After photobleaching, EYFP-HLN1 condensates rapidly recovered (Fig. 3A). Quantification of the fluorescence intensity showed that the half recovery time (T_1/2_) was 43.33 s (Fig. 3B). These data demonstrate that the EYFP-HLN1 protein could undergo LLPS under osmotic stress to form dynamic condensates in vivo.

To investigate whether HLN1 also undergoes phase separation in vitro, we purified HIS-EYFP-HLN1 and HIS-EYFP proteins. Under osmotic stress induced by polyethylene glycol 8000 (PEG8000), only the HIS-EYFP-HLN1 protein, but not HIS-EYFP, formed spherical droplets (Fig. 3C). The size of HIS-EYFP-HLN1 droplets increased in a HLN1 concentration-dependent manner (Fig. 3D). The FRAP assay further revealed that the EYFP signals from the HIS-EYFP-HLN1 droplet rapidly recovered after photobleaching (Fig. 3E) with a T_1/2_ of 23.33 s (Fig. 3F). These data indicate that HIS-EYFP-HLN1 molecules can diffuse freely within the droplets. Taken together, our in vivo and in vitro assays indicate that the HLN1 protein undergoes LLPS to form cytoplasmic granules under osmotic stress conditions.

### Reduced *GAD2 *levels and GABA content in *hln1* under drought stress

The severe drought-sensitive phenotype of the *hln1* mutant suggests that HLN1 regulates key processes in plant drought responses. As an mRNA-binding protein, HLN1 likely influences the metabolism of specific mRNAs critical for stomatal closure during drought stress. To identify the RNA targets, we conducted RIP-seq (RNA-binding protein immunoprecipitation-RNA sequencing) assays, yet the library construction was unsuccessful for unclear reasons. We thus resorted to alternative approaches to identify potential HLN1 targets.

We performed an in-silico analysis of potential RNA targets of HLN1 using RNA–Protein Interaction Prediction (RPISeq) (Muppirala et al. 2011). This analysis predicted hundreds of HLN1-binding mRNAs. Gene Ontology (GO) enrichment analysis showed that these predicted targets were primarily associated with ‘membrane-bounded organelle (GO:0043227)’, ‘intracellular membrane-bounded organelle (GO:0043231)’ and ‘intracellular organelle (GO:0043229)’ (Supplemental Fig. 7 A). Kyoto Encyclopedia of Genes and Genomes (KEGG) enrichment analysis showed that the top pathways include ‘mRNA surveillance pathway’, ‘DNA replication’ and ‘Aminoacyl-tRNA biosynthesis’ (Supplemental Fig. 7B).

While the biological relevance of these pathways in *hln1* remains unclear, we focused on high-probability individual transcripts that may interact with HLN1. By integrating prediction scores with single cell expression data from Arabidopsis Leaf Time-Dependent Atlas (AraLeTA) and leaf transcriptomics (Vong et al. 2024; Tenorio et al. 2025), we identified *GAD2* as a top candidate (Supplemental Figs. 7 C and 7D). *GAD2* encodes one of the glutamate decarboxylases that catalyze the decarboxylation of glutamate to generate γ-aminobutyric acid (GABA), a non-proteinogenic amino acid known to regulate stomatal movement (Mekonnen et al. 2016; Xu et al. 2021).

In Arabidopsis, the glutamate decarboxylase (GAD*)* family comprises five members. The affinity score of *GAD2* to HLN1 was the highest among members (Supplemental Fig. 7 F). In leaf tissues, *GAD2* was the most abundant, being at least 40-fold higher than other *GAD* members (Supplemental Fig. 7G). This is consistent with a previous report identifying *GAD2* as the predominant *GAD* transcript in Arabidopsis leaves (Miyashita and Good 2008). Under drought treatment, we found that transcript levels of *GAD2* in *hln1* mutants were only 63% of wild-type levels (see below, Fig. 4F), suggesting that HLN1 maintains *GAD2* expression during drought stress. Consistent with this finding, *hln1* mutants accumulated significantly less GABA than the wild type under drought stress (Supplemental Figs. 7E).

Since abscisic acid (ABA) synthesized under abiotic stress triggers stomatal closure, we also examined the response of *hln1* towards ABA. While ABA induced the closure of stomata in both the wild type and the *hln1* mutant, there were no significant differences in stomatal apertures between them (Supplemental Figs. 8 A and 8B). Furthermore, drought-induced ABA levels were similar between the *hln1* mutant and the wild type (Supplemental Fig. 8 C). Thus, the impaired stomatal closure of *hln1* under drought stress may not be caused by ABA biosynthesis or signaling defects.

### HLN1 binds and stabilizes *GAD2* transcripts

Based on the above findings, we hypothesized that HLN1 regulates drought responses through post-transcriptional modulation of *GAD2* mRNA levels. To test this hypothesis, we first investigated HLN1–GAD2 interactions using both in vivo and in vitro approaches. RIP-qPCR assays performed with EYFP-HLN1 transgenic seedlings demonstrated that HLN1 preferentially bound to *GAD2* transcripts over *EYFP* transcripts under normal conditions, with significantly enhanced enrichment of *GAD2* transcripts following dehydration treatment (40% fresh weight loss) (Fig. 4B). Electrophoretic mobility shift assay (EMSA) confirmed direct interaction, showing specific binding of MBP-HLN1 to the *GAD2* 3’UTR*,* while the control protein MBP-GST showed no binding activity (Fig. 4C). Interestingly, in vitro assays revealed that *GAD2* mRNA promoted HLN1 condensate formation but failed to form condensates with the control YFP protein (Supplemental Fig. 9).

To evaluate the functional consequences of the interaction between HLN1 and *GAD2* transcripts, we examined the *GAD2* mRNA stability in the *hln1* mutant background. Seven-day-old seedlings were treated with cordycepin to inhibit transcription, and the dynamics of *GAD2* mRNA was monitored over time. It was found that *GAD2* mRNA levels decreased much faster in the *hln1* mutant than in the wild type (Fig. 4D). Conversely, *GAD2* mRNA in the two independent *HLN1* overexpression lines were more stable than in the wild type (Fig. 4E). These data indicate that HLN1 could stabilize *GAD2* mRNA, protecting the transcripts from rapid degradation.

We measured the expression levels of *GAD2* in two independent complementation lines generated by transforming the *hln1* mutant with the wild type *HLN1* genomic DNA under the control of its native promoter (Sajeev et al. 2022). Under control conditions, the expression level of *GAD2* was similarly low among all genotypes. Drought stress increased *GAD2* expression, yet the expression level in *hln1* was significantly lower than in the wild type (Fig. 4F). Meanwhile, the transcript levels of *GAD2* in the two complementation lines were restored to levels comparable to the wild type. Furthermore, *HLN1-*overexpression lines showed elevated *GAD2* transcript levels under drought conditions (Supplemental Figs. 3 F and 3G).

GAD2 is a key enzyme for GABA synthesis in leaves (Miyashita and Good 2008) and altered *GAD2* mRNA levels would affect plant GABA content (Mekonnen et al. 2016). We quantified GABA levels across genotypes. While all lines showed similar baseline GABA content under control conditions, significant differences were found under drought stress. *hln1* mutants accumulated less GABA than wild-type plants, while both complementation lines restored GABA production to wild-type levels (Fig. 4G). *HLN1* overexpression plants exhibited even high GABA accumulation (Supplemental Fig. 3G). Thus, there is a clear correlation among HLN1 dosage, *GAD2* transcript levels, and GABA accumulation under drought stress. These results establish that HLN1 stabilizes *GAD2* transcripts through direct binding and leads to enhanced GABA production during drought stress. The formation of HLN1-*GAD2* mRNA condensates likely represents a key mechanistic step in GABA-mediated stomatal responses to drought stress.

### HLN1 regulates stomatal movement through GABA signaling

Recent studies have established GABA as a signaling molecule that triggers stomatal closure during drought stress (Mekonnen et al. 2016; Xu et al. 2021, 2024). To test whether HLN1 enhances drought tolerance by regulating *GAD2* mRNA stability and consequently modulating stomatal movement through GABA signaling, we examined the effect of exogenous GABA on stomatal responses in the *hln1* mutant. Three-week-old plants of Col-0, *hln1*, and two HLN1 complementation lines were sprayed with 4 mM GABA and subjected to 10 days of water withholding before stomatal aperture measurement.

Under well-watered conditions, GABA treatment showed no significant effect on stomatal movement. However, during drought stress, we observed two key findings: 1) wild type plants showed reduced stomatal apertures that were further enhanced by GABA application (Fig. 5A-B), and 2) exogenous GABA completely rescued the impaired stomatal closure phenotype of *hln1* mutants (Fig. 5A-B). These observations are consistent with previous reports that GABA promotes stomatal closure and enhances drought tolerance (Xu et al. 2021) and indicate that HLN1 regulates stomatal movement at least partially through GABA-mediated pathways.

We further characterized the physiological effects of GABA treatment by measuring stomatal conductance, reactive oxygen species (ROS) accumulation, and lipid peroxidation levels. GABA application significantly reduced all three parameters in *hln1* mutants compared to untreated controls (Fig. 5C-E). These data demonstrate that these drought-stress related phenotypic defects in the *hln1* mutant can be rescued by exogenous GABA treatment.

### Overexpression of *GAD2* rescues drought sensitivity in *hln1*mutants

**Fig. 6:**
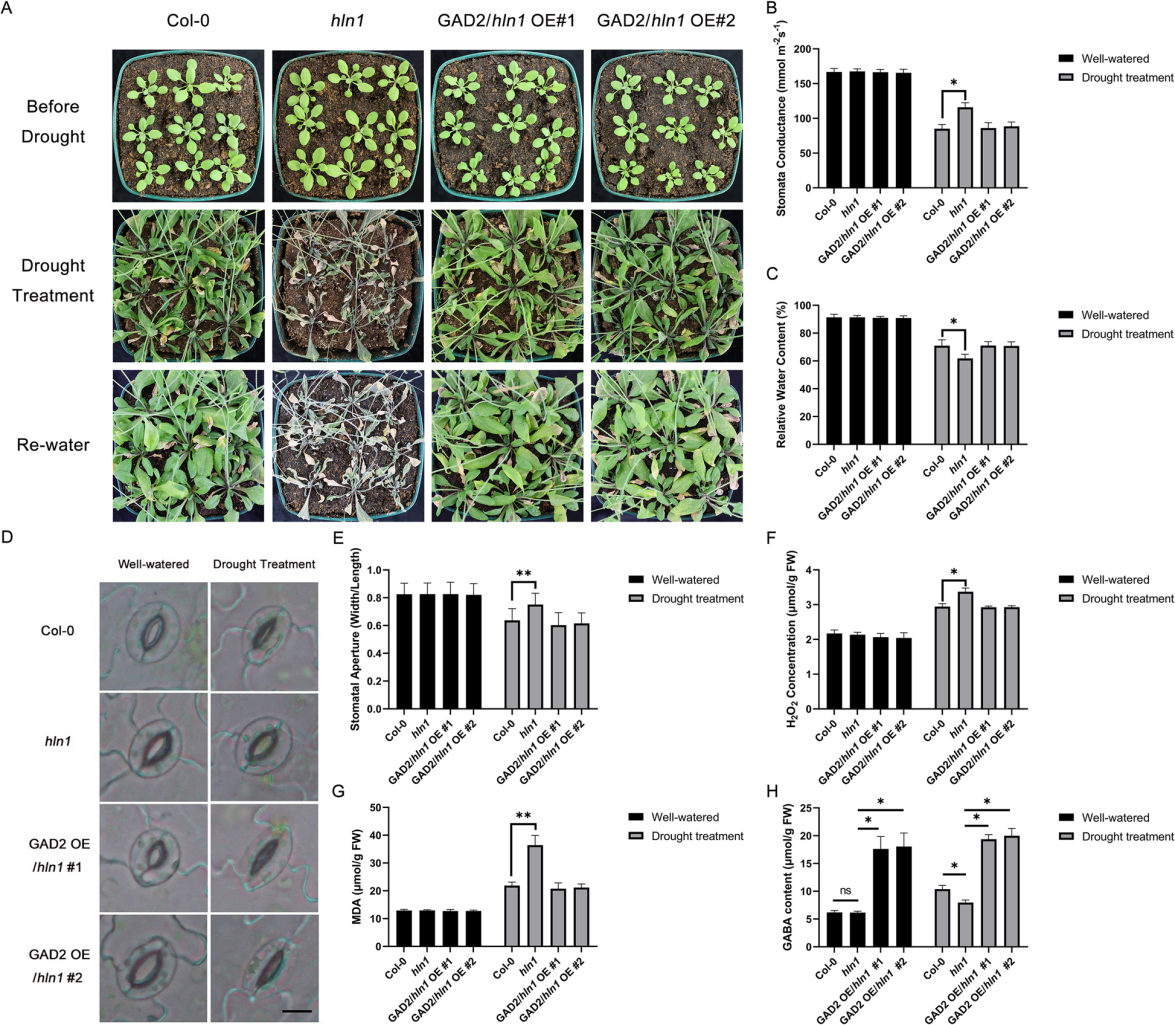
Overexpression of *GAD2* enhances drought tolerance of the *hln1* mutant. **A** Morphology of 3-week-old plants of the indicated genotypes before (upper panel) and after (middle panel) 20-day drought stress treatment, and 2 days after rewatering (lower panel). **B** Stomatal conductance in rosette leaves after drought treatment. **C** Relative water content (RWC) in rosette leaves after 10-day drought treatment. **D** Stomatal morphology in rosette leaves under drought treatment. Leaves were excised and imaged immediately with a light microscope after 10-day drought treatment. More than 100 stomata of each genotype were measured, and the experiments were repeated three times with similar results. Scale bar = 10 μm. **E** Stomatal apertures (expressed as width/length ratio) under control or drought treatment. Experiments were repeated three times and stomatal apertures from more than 100 stomata were calculated. **F** Hydrogen peroxide (H_2_O_2_) content in rosette leaves under control or drought treatment. **G** Malondialdehyde (MDA) content in rosette leaves under control or drought treatment. **H** GABA content in rosette leaves under control or drought treatment. Plant genotypes include Col-0 (wild type); *hln1* (*hln1* mutant); GAD2/*hln1* OE #1 and #2 (two independent *hln1* complementation lines). Data in (**B**-**C** and **F**-**G**) are means and standard deviation (SD) from three biological replicates. The double asterisks (**) indicate a *p*-value < 0.01, and the single asterisk (*) indicates a *p*-value < 0.05 by Student’s *t*-test

To determine whether HLN1-sustained *GAD2* transcript stability contributes to drought tolerance, we introduce a *35S:Flag-GAD2* construct into *hln1* mutants and assessed their drought responses. As shown in Fig. 6A, overexpressing *GAD2* effectively rescued the drought-induced seedling lethality observed in *hln1* mutants. Notably, both *GAD2*/*hln1* overexpressing (OE) lines accumulated endogenous GABA at levels more than double those of the wild type plants (Fig. 6).

Compared with the *hln1* mutant, the stomatal conductance and stomatal aperture in both *GAD2*/*hln1* OE lines decreased under drought stress (Fig. 6B and D-E), leading to the restoration of RWC to wild-type levels (Fig. 6C). Consistently, relative to the *hln1* mutant, the content of H_2_O_2_ and MDA was reduced in the *GAD2*/*hln1* OE lines (Fig. 6F-G), suggesting less oxidative stress in these plants under drought conditions. These findings demonstrate that *GAD2* overexpression compensates for HLN1 loss-of-function and support our model in which HLN1 enhances drought tolerance through stabilization of *GAD2* transcripts thereby maintaining adequate GABA levels for proper drought responses and tolerance.

## Disscussion

Recent studies have identified the Arabidopsis HLN1 protein as part of an RNA-binding proteome that inhibits seed germination through mechanisms that remain unclear (Sajeev et al. 2022). Our current research demonstrates HLN1's critical role in plant drought stress tolerance. Under osmotic stress conditions, HLN1 undergoes liquid–liquid phase separation (LLPS) to form cytoplasmic condensates, interacting with stress-related RNAs such as *GAD2*. These ribonucleoprotein (RNP) condensates may stabilize *GAD2* transcripts, thereby increasing GABA levels and enhancing drought tolerance.

As an RNA-binding protein (RBP), HLN1 contains a conserved HABP4 domain and an RGG repeat motif, both are recognized RNA-binding regions (Thandapani et al. 2013; Hentze et al. 2018) and are conserved across the three-membered Arabidopsis HLN1 family (AtRGGA/At4 g16830, AtRGGB/AT4G17520, and HLN1/AtRGGC/AT5G47210) (Ambrosone et al. 2015; Sajeev et al. 2022; Bleckmann et al. 2023). Additionally, HLN1 possesses multiple intrinsically disordered regions (IDRs) that drive LLPS (Hentze et al. 2018). Our assays confirmed HLN1's ability to form dynamic, liquid-like membraneless condensates under conditions such as higher osmolarity and cellular crowding (Fig. 3A and C; Supplemental Fig. 9). This behavior is consistent with other RNA-binding proteins (Alshareedah et al. 2020; Zhu et al. 2022; Fan et al. 2024; Wadsworth et al. 2024; Wang et al. 2024) and suggests that HLN1's functions are likely mediated by these dynamic RNP condensates.

Previous reports have indicated that AtRGGA enhances salt and drought tolerance (Ambrosone et al. 2015), though the mechanisms were unknown. Our analyses identified *GAD2* as a candidate mRNA target of HLN1, confirmed by EMSA and RIP-qPCR assays. Notably, *GAD2* is the most abundant GAD family member in Arabidopsis leaves that controls GABA production (Miyashita and Good 2008). HLN1-*GAD2* condensates likely stabilize *GAD2* transcripts, leading to higher GABA levels under drought conditions. This functionality of *HLN1* is similar to that of rice DRG9, which stabilizes *OsNCED4* mRNA to enhance ABA biosynthesis and drought tolerance (Wang et al. 2024). Our pharmacological inhibition experiments demonstrated faster decay of *GAD2* mRNA in *hln1* mutants and increased stability with HLN1 overexpression. This stabilization correlates with enhanced drought tolerance, as evidenced by the recovery of drought tolerance in *hln1* mutants through *GAD2* overexpression or GABA application (Fig. 6A).

In recent years, GABA has been found to regulate stomatal movement under drought stress (Mekonnen et al. 2016; Xu et al. 2021), in addition to its diverse functions in biotic and abiotic stress responses (Islam et al. 2024). GABA interacts with aluminum-activated malate transporters (ALMTs) and modulates the malate uptake into the vacuoles of guard cells, which blocks the opening of stomata and enhances drought tolerance (Ramesh et al. 2015; Gilliham and Tyerman 2016; Xu et al. 2021). Upon GABA deficiency or depletion under drought stress, the stomatal closure would be impaired. This is consistent with our data that loss of function in *HLN1* leads to reduced GABA contents and greater stomatal aperture under drought stress (Fig. 1D-E). As the primary enzyme for GABA synthesis in leaves, both the transcriptional and posttranscriptional regulation of *GAD2* would be important for GABA production.

**Fig. 7:**
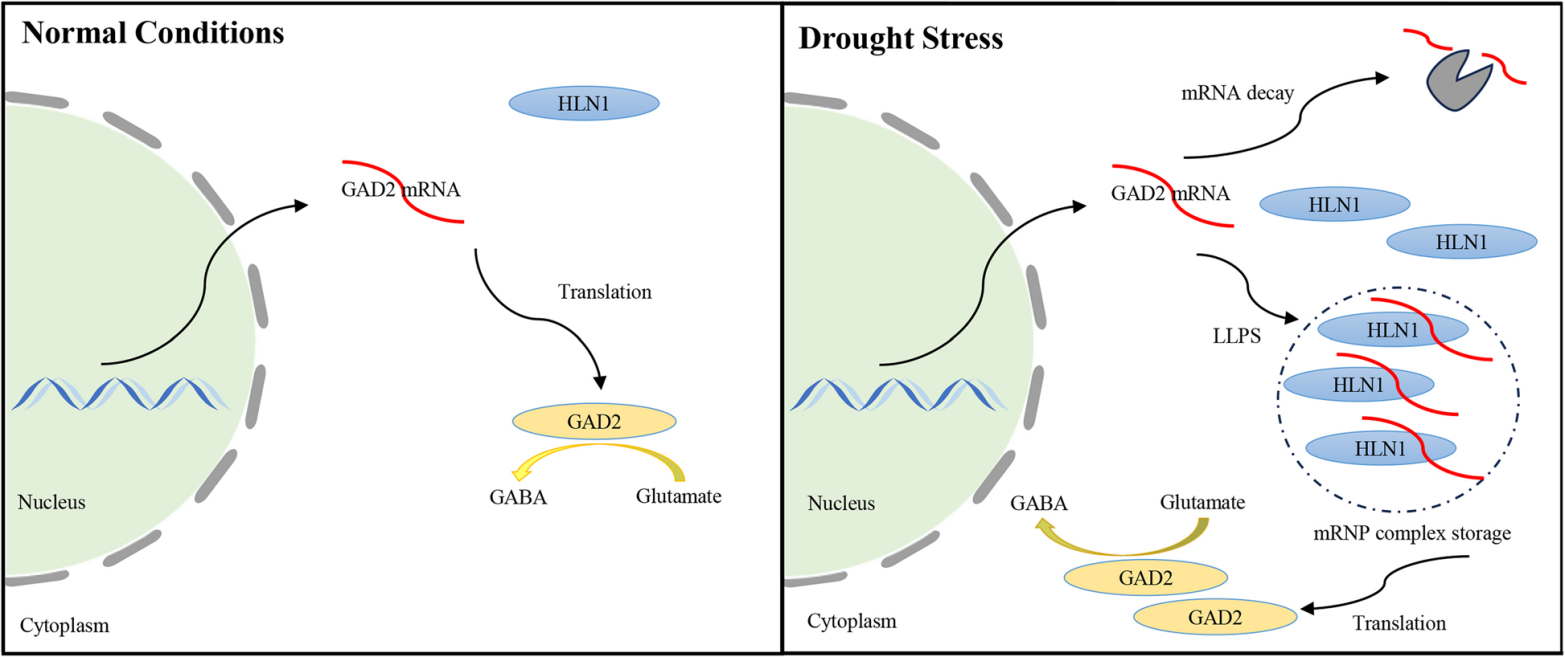
HLN1 condensates stabilize *GAD2* mRNA during drought stress. Glutamate decarboxylase 2 (GAD2) catalyzes the conversion of glutamate to γ-aminobutyric acid (GABA) in leaves. Under well-watered conditions, *GAD2* transcript levels remain low, supporting basal GABA synthesis. During drought stress, *GAD2* expression increases. HLN1 binds with *GAD2* mRNA and other transcripts and undergoes liquid–liquid phase separation (LLPS) to form the mRNA-protein (mRNP) condensates that protect *GAD2* mRNA from rapid degradation. This stabilization enables sustained and elevated GAD2 protein production, enhancing GABA synthesis under drought conditions. In the absence of *HLN1*, *GAD2* mRNA becomes less stable, reducing GABA levels and impairing stomatal closure during drought stress

We propose a model where HLN1 enhances drought tolerance by stabilizing *GAD2* mRNA, thereby promoting GABA synthesis and facilitating stomatal closure through interacting with ALMTs (Fig. 7). This mechanism appears to be crucial for maintaining GABA levels and stomatal regulation during drought stress. While transcriptional regulation of stress genes is well-documented, the role of posttranscriptional regulation including mRNA stabilization via RBPs is less explored. Previous transgenic approaches to regulate plant stress resistance are also more focused on regulation of transcription through transcription factor manipulations, yet these approaches often result in reduced or stunt growth of the transgenic plants (Gong et al. 2020). In our current study, we did not notice any obvious phenotypic changes of transgenic plants overexpressing *HLN1* under normal conditions (Supplementary Fig. 3). Thus, targeting mRNA stability through RBPs like HLN1 presents a promising strategy for engineering drought-resistant crops without yield penalties.

### Limitations of the study

While our study provides insights into HLN1's role in drought tolerance, the current work has several limitations. First, the identification of HLN1’s full mRNA target repertoire remains incomplete. While *GAD2* was confirmed as a key target, HLN1 likely binds other transcripts that contribute to drought tolerance or other physiological processes. Second, the precise roles of HLN1’s IDRs in condensate formation and RNA binding are unclear. Our initial mutational analyses of individual IDR functions in RNA-binding and condensate formation were challenging and more sophisticated approaches to dissect IDR functionality are needed. Third, this study focused primarily on GABA-mediated stomatal regulation, but HLN1’s role in other drought-related processes, such as reactive oxygen species (ROS) suppression (Li et al. 2021; Xu et al. 2024), remains unexplored. Finally, while our proposed model emphasizes transcript stability, HLN1 may also regulate transcript processing and translation, as suggested by its homologs in other species (Ma et al. 2020). These processes could potentially impact GAD2 protein levels or enzymatic activities and warrant further investigation in order to fully understand HLN1's role in stress adaptation.

## Conclusions

Our study establishes HLN1 as a critical drought tolerance regulator that stabilizes *GAD2* mRNA, enhancing GABA production, and regulating stomatal closure during drought stress. This research provides insights into posttranscriptional regulation of stress genes and underscores the significance of RBPs in plant stress responses. Targeting mRNA stability through RBPs like HLN1 offers promising strategies for engineering drought-resistant crops without compromising yield. Further studies on HLN1 and other RBPs may reveal novel mechanisms of plant adaptation to environmental stresses and identify new targets for improving stress tolerance.

## Materials and methods

### Plant materials and growth conditions

The *Arabidopsis thaliana* ecotype Col-0, referred to as the wild type, was used in this study. The *hln1* knockout mutant (SALK_055953) and its complementation lines were generously provided by Professor Leonie Bentsink (Sajeev et al. 2022). For growth assays, seeds were surface-sterilized in 75% ethanol for 10 min and then rinsed five times with sterile Milli-Q water. The sterilized seeds were sown on ½-strength Murashige and Skoog (MS) medium plates containing 1% agar and 1% sucrose. The plates were incubated at 4 ℃ for 3 days before being transferred to a plant growth room maintained at 22 ℃ with a 16-h light/8-h dark cycle.

### Drought treatment and relative water content (RWC) measurement

Seven-day-old seedlings were transferred to pots containing a uniform amount of soil (250 g) with 9 seedlings per pot. For drought treatment, 3-week-old plants were subjected to a 20-day period without watering, followed by rewatering. The morphology of the plants was documented using a Canon EOS R7 camera at the beginning and at the end of the drought treatment and at 2 days after rewatering.

Relative water content (RWC) was calculated using the following equation: RWC = (FW-DW)/(SW-DW), where FW represents fresh weight, DW represents the dry weight and SW represents saturated weight.

### Stomatal aperture, conductance, and density

For stomatal aperture analysis, the fifth or sixth pair of rosette leaves from 3-week-old plants was excised, and epidermal peels were immediately prepared for imaging under a light microscope equipped with a digital camera. Stomatal apertures were measured using ImageJ software (NIH, USA).

Stomatal conductance was measured using a METER SC-1 Leaf Porometer (Team Medical & Scientific Sdn. Bhd., Malaysia). The instrument was first calibrated according to the manufacturer’s instructions prior to measurements. The sensor was clipped onto the leaves, and readings were recorded after stabilization.

Stomatal density was determined by photographing leaf epidermis peels. Specifically, the fifth or sixth pair of rosette leaves from 3-week-old plants was excised, and epidermal peels were immediately mounted on microscope slide for observation under a light microscope equipped with a digital camera. Stomata were counted under each documented view to determine density (number/mm^2^).

### Hydrogen peroxide, MDA and GABA quantification

Leaf hydrogen peroxide (H_2_O_2_) of 3-week-old plants was assayed using a Hydrogen Peroxide Assay Kit (BC3595, Solarbio Co., Beijing, China) following the manufacturer’s instruction.

Malondialdehyde (MDA) content was measured using the method described by Hodges et al. (1999). Briefly, 0.5 g of fresh plant samples was homogenized in 2 mL of chilled 10% TCA (trichloroacetic acid) buffer. After centrifugation at 10,000 g for 15 min, the supernatant was mixed with an equal volume of 0.6% thiobarbituric acid (TBA). The mixture was then heated at 100 ℃ for 20 min, cooled on ice, and centrifuged again at 10,000 g for 10 min. The supernatant was collected and the absorbance measurement at 532 nm, 600 nm and 450 nm were obtained using a Shimadzu UV-1800 spectrophotometer. The MDA content was calculated according to the formula: 6.45 × (A_532_ – A_600_) – 0.56 × A_450_.

GABA content was determined using the Elabscience® GABA Colorimetric Assay Kit (E-BC-K852-M, Elabscience) according to the manufacturer’s instructions. In brief, leaf samples from 3-week-old plants, with or without 10-day water-withheld treatment, were ground with the extraction solution. The supernatant was reacted with phenol and sodium hypochlorite to produce a blue-green product that has a maximum absorbance at 640 nm. The GABA content was calculated based on absorbance of samples in comparison with the standard curves.

### ABA content and ABA-induced stomatal closure

ABA content was measured using an Abscisic acid (ABA) ELISA Kit (Fankew Industrial Co., Ltd., Shanghai). Briefly, leaves from 3-week-old Arabidopsis plants, with or without a 10-day drought treatment, were harvested for ABA quantification following the manufacturer's protocol. Absorbance was measured using a MULTISKAN GO Microplate Reader (Thermo).

For stomatal aperture assays, leaves from 3-week-old Col-0 and *hln1* plants were excised and immersed in a Leaf Open Buffer (10 mM MES-Tris (pH 5.6), 5 mM KCl, 50 μM CaCl2) under light for 2 h, followed by transfer to the same containing 25 μM ABA. After 2 h incubation, epidermal peels were prepared and imaged using an Olympus CKX41 microscope.

### Plant transformation

For HLN1 subcellular localization, *HLN1* cDNA was amplified and cloned into the pDONR™/Zeo vector (Invitrogen) using Gateway™ BP Clonase™ II Enzyme mix (Cat. # 11,789,020, Thermo). The EYFP-tagged destination vectors, including pEarleyGate 101-HLN1 or pGWB541-HLN1 for C-terminal tagging and pEarleyGate 104-HLN1 or pGWB542-HLN1 for N-terminal tagging, were generated via Gateway™ LR Clonase™ II Enzyme mix (Cat. # 11,791,020, Thermo). For *GAD2* overexpression in *hln1*, *GAD2* cDNA was cloned into the pDONR™/Zeo vector. The destination vector pEarleyGate 202-GAD2 was obtained through LR recombination. Transgenic plants were generated using *Agrobacterium tumefaciens* (strain GV3101) -mediated floral dip transformation. All primers are listed in Supplemental Table 2.

### GUS staining

The 1677 bp promoter of *HLN1* was amplified from Arabidopsis genomic DNA and cloned into the pDONR-HLN1 vector. The pGWB433-pHLN1 vector was obtained via LR reaction. Transgenic plants were generated using the floral dip transformation method. GUS staining was performed with T_3_ generation seedlings or adult plants using GUS Stain solution (BN20175, Biorigin) according to the manufacturer’s instructions. Tissues were incubated in the X-gluc solution at room temperature overnight, then destained in ethanol prior to observation.

### Subcellular localization

Confocal images were acquired using a Leica confocal microscope. The subcellular localization of HLN1 was determined in root tips from 7-day-old transgenic seedlings. Transgenic plants expressing either N-terminal EYFP tagged and C-terminal EYFP tagged HLN1 fusion proteins were examined, and similar results were obtained. For mannitol treatment, 7-day-old seedlings were incubated in 0.4 M mannitol solution for 30 min. For CHX treatment, 7-day-old seedlings were first incubated with 50 μM CHX for 30 min at room temperature and then transferred to 0.4 M mannitol solution for an additional 30 min before observation.

### Fluorescence recovery after photobleaching (FRAP)

For in vivo FRAP experiments on the EYFP-HLN1 condensates in root elongation zone cells, we used a Leica Stellaris 5 confocal microscope with a × 63 objective lens. EYFP-HLN1 condensates were bleached using a 488-nm pulse at 50% intensity. Fluorescence recovery was recorded every 10 s for up to 160 s post-bleaching. Images were acquired and quantified using the LAS X software.

For in vitro FRAP analysis of HIS-EYFP-HLN1 droplets, the central area of selected droplets was bleached using a 488-nm pulse at 50% intensity. Fluorescence recovery was similarly monitored every 10 s for up to 160 s post-bleaching. To calculate the recovery rate, the fluorescence values were normalized to the first time point and expressed as a percentage of the pre-bleach intensity.

### Protein expression and purification

To construct the pET28a-EYFP-HLN1 and pET28a-EYFP plasmids, the EYFP-HLN1 and EYFP fragments were cloned from the pGWB542-HLN1 plasmid and inserted into the pET28a plasmid using the ClonExpress II One Step Cloning Kit (C112, Vazyme) at the EcoRI and HindIII sites. For the MBP-HLN1 plasmid, the HLN1 insert was cloned into the pMAL-c5x plasmid at the SalI site via homologous recombination. For prokaryotic expression, the plasmids were expressed in the *E. coli* BL21 (DE3) strain and proteins were purified with Pierce™ Glutathione Magnetic Agarose Beads (78,601, Thermo) or Amylose Magnetic Beads (E8035S, NEB) according to the manufacturer’s instructions.

### EMSA analysis

To generate the 3’UTR and full-length mRNA of *GAD2* via in vitro transcription, the *GAD2* 3’UTR and the full-length cDNA were synthesized and inserted into the pcDNA3.1(+) plasmid driven by a T7 promoter. The 3’UTR and full-length mRNA transcripts were then synthesized using the T7 High Yield RNA Transcription Kit (TR101-01, Vazyme) and were purified using the Monarch RNA Cleanup Kit (T2040S, NEB). The protein-mRNA interaction assays were performed as previously described (Seo et al.^ 2019^). Briefly, mRNA was incubated with the MBP-HLN1 protein at room temperature for 30 min and analyzed by native PAGE gel. RNA shift signals were detected using Ultra GelRed Nucleic Acid Stain (GR 501–01, Vazyme).

### RNA isolation and real-time PCR

Total RNA was extracted from 3-week-old plants using RNeasy Plant Mini Kit (74,904, Qiagen) along with the RNase-Free DNase Set (79,254, Qiagen). cDNA synthesis was performed using the HiScript II 1 st Strand cDNA Synthesis Kit (R211-01, Vazyme). Real-time qPCR reactions were performed using the ABI PCR System with ChamQ Universal SYBR qPCR Master Mix (Q711-02, Vazyme), using 18S rRNA as the internal control. Three biological replicates were performed, and the standard deviation (SD) was calculated. Primers used in this study are listed in Supplemental Table 1.

### mRNA decay analysis

Seven-day-old seedlings were incubated with 1 mM cordycepin (HY-N0262, MCE) and vacuum infiltrated for 15 min. After the treatment, samples were harvested at specific time points. Three biological replicates were performed, each using approximately 0.5 g of sample. Total RNA was isolated for subsequent qRT-PCR analysis, using *UBQ5* as the internal control. Primers used for qRT-PCR are listed in Supplemental Table 1.

### RIP-qPCR analysis

The RIP assay was conducted similarly as described (Song et al. 2023). Briefly, 14-day-old seedlings were harvested, and approximately 3-g of sample were treated with 30 mL of 1% formaldehyde solution under vacuum for 15 min. The fixation reaction was quenched using 2 M glycine for 5 min under vacuum. The samples were then washed three times with water and blotted on paper towels to remove excess water. Subsequently, the samples were frozen with liquid nitrogen and ground into powder. Lysis buffer was added to powdered samples, and the mixture was incubated at 4 ℃ for 30 min. After centrifugation, half of the lysate were saved as input, while the remaining lysate was incubated with anti-GFP magnetic beads (P2132, Beyotime Biotech Inc, Shanghai, China) at 4 ℃ for 2 h with rotation. Following beads washing and proteinase K treatment, RNA was extracted using Trizol reagent. The relative enrichment of RNA was determined by qRT-PCR. For the dehydration treatment, seedlings were exposed to air until 40% of their fresh weight was lost. The primers used for this assay are listed in Supplemental Table 1.

### In silico RIP-seq analysis

Transcripts from Arabidopsis Col-0 plants were obtained from the Ensembl database, and the HLN1 protein sequence was retrieved from the NCBI database. These sequences were analyzed using the RPIseq web server (http://pridb.gdcb.iastate.edu/RPISeq/) in batch submission mode. A cutoff threshold of > 0.95 for both the random forest (RF) and support vector machine (SVM) classifiers was applied to identify positive hits. The interacting candidates were ranked based on gene expression data in Arabidopsis leaves (Vong et al. 2024; Tenorio et al. 2025). Functional annotation of the positive hits was performed using Gene Ontology (GO) and KEGG pathway analyses via the OmicShare platform (Mu et al. 2024).

### Functional domain and intrinsically disordered region analysis

The functional domains of HLN1 were analyzed using InterPro (Blum et al. 2025). The intrinsically disordered regions (IDRs) of the HLN1 protein were predicted using PONDR (Peng et al. 2006). Illustrations were generated using GraphPad.

## Supplementary Information

Supplementary Material 1. Supplemental Figure 1. Stomatal density in rosette leaves of Col-0 and *hln1*. Supplemental Figure 2. Relative *HLN1* expression in *HLN1* overexpression lines. Supplemental Figure 3. Overexpression of *HLN1* confers drought tolerance. Supplemental Figure 4. Relative expression of *HLN1* in different tissues. Supplemental Figure 5. Expression of *HLN1* was induced by drought stress. Supplemental Figure 6. Intrinsically disordered region (IDR) domain analysis of HLN1. Supplemental Figure 7. In-silico analysis of candidate targets of HLN1. Supplemental Figure 8. Stomatal response to ABA and ABA content of the wild type and *hln1* mutant under drought stress. Supplemental Figure 9. HLN1 condensates with *GAD2* mRNA in vitro. Supplemental Figure 10. Functional domain analysis of HLN1. Supplemental Table 1. Primers used in genotyping and qRT-PCR analysis. Supplemental Table 2. Primers used to construct plasmids.

## Data Availability

All data supporting the findings of this study are included in the article and its supplementary materials.

## Abbreviations

ABA: Abscisic acid
ALMT: Aluminum-activated malate transporter
AraLeTA: Arabidopsis Leaf Time-Dependent Atlas
AtRGGA/B/C: Arabidopsis RGG (Arg-Gly-Gly) motif protein A/B/C
CHX: Cycloheximide
DRG9: Drought resistance gene 9
DW: Dry weight
EMSA: Electrophoretic mobility shift assay
EYFP: Enhanced yellow fluorescent protein (YFP)
FRAP: Fluorescence recovery after photobleaching
FW: Fresh weight
GABA: γ‐Aminobutyric acid
GAD1/2: Glutamate decarboxylase 1/2
GFP: Green fluorescent protein
GO: Gene Ontology
GUS: β-Glucuronidase
HABP4/SERBP1: Hyaluronan-binding protein 4 / serpin mRNA binding protein 1
HIS: Histidine
HLN1: Hyaluronan 1
IDR: Intrinsically disordered region
KEGG: Kyoto Encyclopedia of Genes and Genomes
LLPS: Liquid–liquid phase separation
LOT1: Low Temperature 1
MDA: Malondialdehyde
mRNP: mRNA-ribonucleoprotein
MS: Murashige and Skoog (salt)
OsNCED4: Rice 9-*ci*s-epoxycarotenoid dioxygenase 4
PAGE: Polyacrylamide gel electrophoresis
P-bodies: Processing bodies
PEG: Polyethylene glycol
qRT-PCR: Quantitative RT-PCR
RBP: RNA-binding protein
RF: Random forest
RGG: Arg-Gly-Gly (motif)
RIP-qPCR: RNA-binding protein immunoprecipitation-quantitative RT-PCR
RIP-seq: RNA-binding protein immunoprecipitation-RNA sequencing
ROS: Reactive oxygen species
RPISeq: RNA–Protein Interaction Prediction
RWC: Relative water content
SD: Standard deviation
SVM: Support vector machine
SW: (Water) saturated weight
TBA: Thiobarbituric acid
TCA: Trichloroacetic acid
*UBQ5*: *UBIQUITIN 5*
UTR: Untranslated region
X-gluc: 5-Bromo-4-chloro-3-indolyl-β-D-glucuronide
