# Supplementary material for: The mRNA-binding protein HLN1 enhances drought stress tolerance by stabilizing the GAD2 mRNA in Arabidopsis
Source: Stress Biol. 2025 Jun 6;5(1):39. doi: 10.1007/s44154-025-00239-4 (PMC12144001; doi:10.1007/s44154-025-00239-4)
Supplement: Supplementary file 1 — Supplementary Material 1. Supplemental Figure 1. Stomatal density in rosette leaves of Col-0 and hln1. Supplemental Figure 2. Relative HLN1 expression in HLN1 overexpression lines. Supplemental Figure 3. Overexpression of HLN1 confers drought tolerance. Supplemental Figure 4. Relative expression of HLN1 in different tissues. Supplemental Figure 5. Expression of HLN1 was induced by drought stress. Supplemental Figure 6. Intrinsically disordered region (IDR) domain analysis of HLN1. Supplemental Figure 7. In-silico analysis of candidate targets of HLN1. Supplemental Figure 8. Stomatal response to ABA and ABA content of the wild type and hln1 mutant under drought stress. Supplemental Figure 9. HLN1 condensates with GAD2 mRNA in vitro. Supplemental Figure 10. Functional domain analysis of HLN1. Supplemental Table 1. Primers used in genotyping and qRT-PCR analysis. Supplemental Table 2. Primers used to construct plasmids. [file 44154_2025_239_MOESM1_ESM.docx]

**SUPPLEMENTAL INFORMATION**

**Supplementary Figures**

Supplemental Figure 1. Stomatal density in rosette leaves of Col-0 and *hln1*.

Supplemental Figure 2. Relative *HLN1* expression in *HLN1* overexpression lines.

Supplemental Figure 3. Overexpression of *HLN1* confers drought tolerance.

Supplemental Figure 4. Relative expression of *HLN1* in different tissues.

Supplemental Figure 5. Expression of *HLN1* was induced by drought stress.

Supplemental Figure 6. Intrinsically disordered region (IDR) domain analysis of HLN1.

Supplemental Figure 7. In-silico analysis of candidate targets of HLN1.

Supplemental Figure 8. Stomatal response to ABA and ABA content of the wild type and *hln1* mutant under drought stress.

Supplemental Figure 9. HLN1 condensates with *GAD2* mRNA in vitro.

Supplemental Figure 10. Functional domain analysis of HLN1.

**Supplementary Tables**

Supplemental Table 1. Primers used in genotyping and qRT-PCR analysis.

Supplemental Table 2. Primers used to construct plasmids.


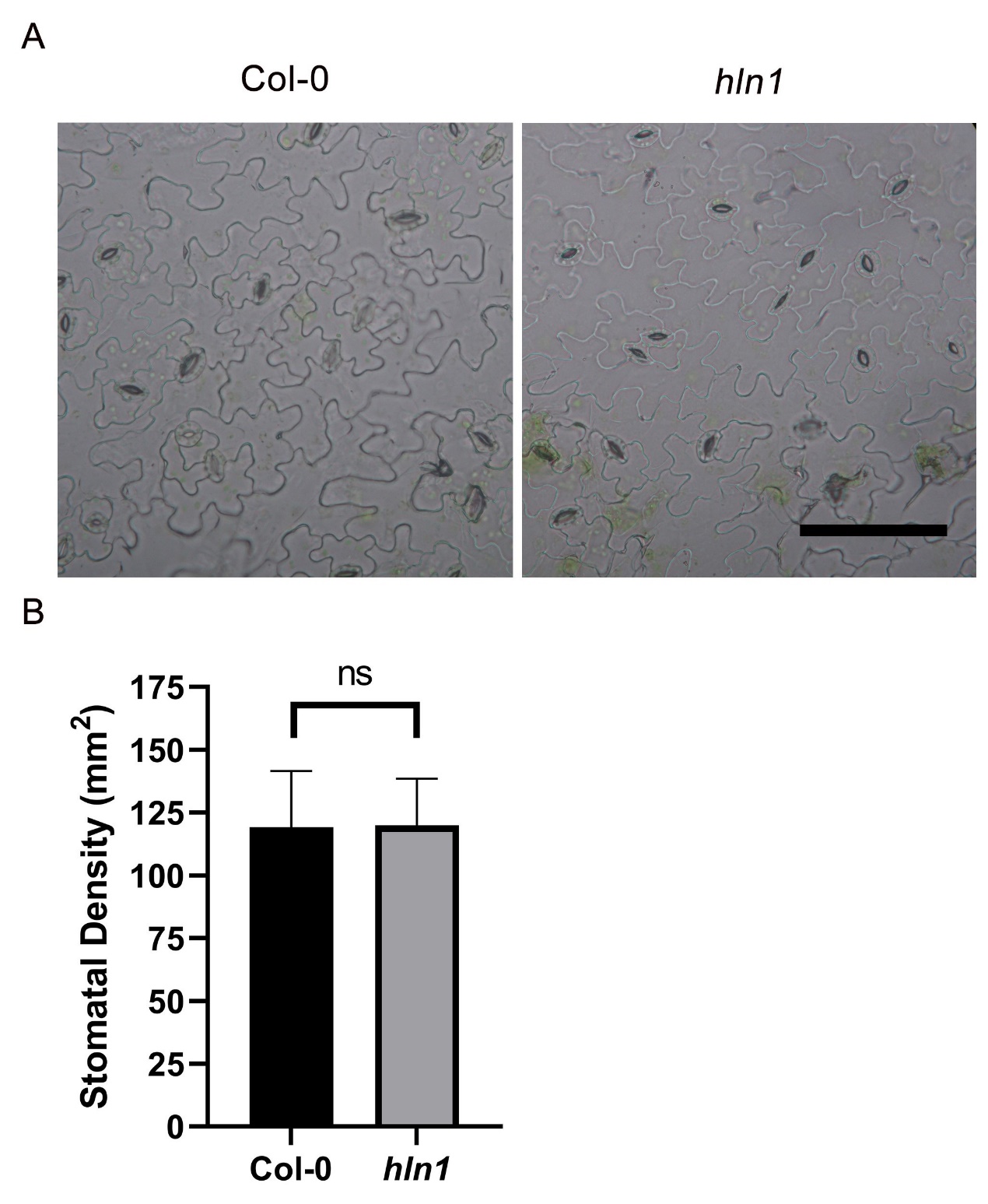


**Supplemental Figure 1**. Stomatal density in rosette leaves of Col-0 and *hln1*.

(A) Representative images of the stomata distribution in the fifth or sixth rosette leaves. Scale bar = 100 µm.

(B) Stomatal density in Col-0 and *hln1*. Assays were repeated three times and more than 500 stomata were counted. Data are means ± standard deviations (SD) from 28 biological replicates. The *p*-value was calculated using Student’s *t*-test.


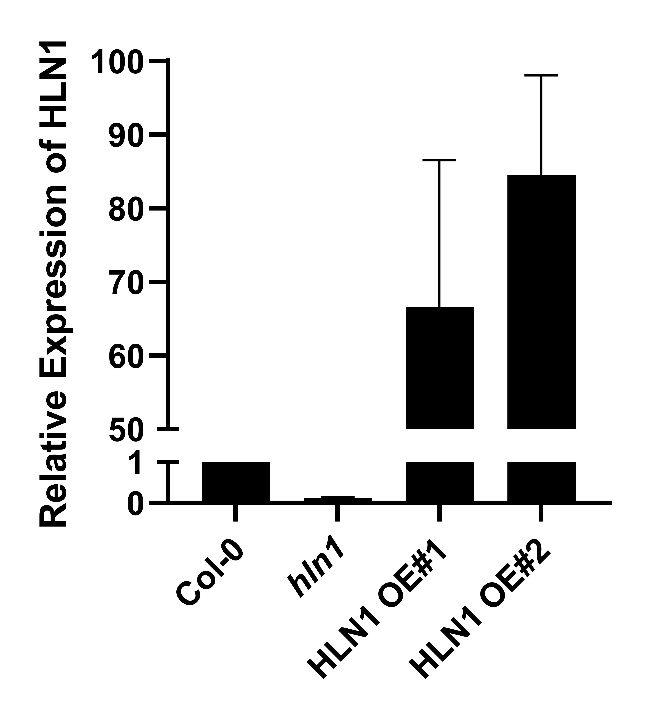


**Supplemental Figure 2**. Relative *HLN1* expression in *HLN1* overexpression lines.

Leaves of two *HLN1* overexpression lines (in the Col-0 background), along with Col-0 and the *hln1* mutant were collected for total RNA extraction and subsequent qRT-PCR analysis. *ACTIN2* was used as an internal control. Data represent means ± standard deviations (SD) of three biological replicates.


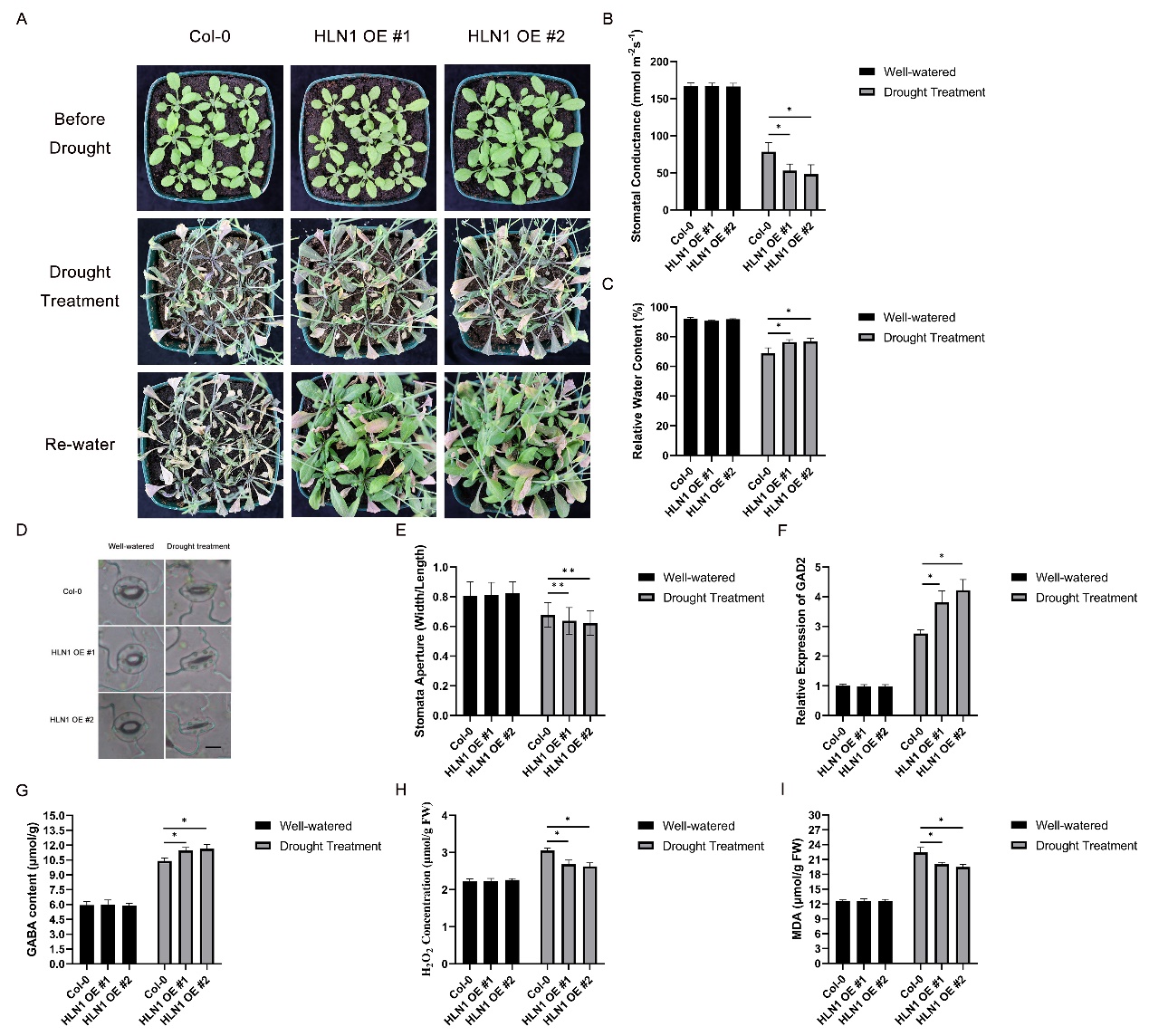


**Supplemental Figure 3**. Overexpression of *HLN1* confers drought tolerance.

(A) Morphology of the wild type (Col-0) and two *HLN1* overexpression lines (HLN1 OE #1 and HLN1 OE #2) before (upper panel), after (middle panel) a 23-day drought stress treatment, and 3 days after rewatering (lower panel).

(B) Stomatal conductance in rosette leaves of 3-week-old plants of Col-0, HLN1 OE #1 and HLN1 OE #2, with or without drought treatment.

(C) Relative water content (RWC) in rosette leaves of 3-week-old plants of Col-0, HLN1 OE #1 and HLN1 OE #2, with or without drought treatment.

(D) Stomatal morphology in rosette leaves of Col-0, HLN1 OE #1 and HLN1 OE #2 plants under drought treatment. Leaves were excised after a 10-day drought treatment and immediately photographed with a light microscope. Scale bar = 10 μm.

(E) Stomatal apertures (expressed as width-to-length ratio) in leaves of Col-0, HLN1 OE #1 and HLN1 OE #2 plants, with or without drought treatment. Experiments were repeated three times and stomatal apertures from more than 100 stomata were calculated. Data represent means ± standard deviations (SD).

(F) Relative expression of *GAD2* mRNA in Col-0, HLN1 OE #1 and HLN1 OE #2 plants, with or without drought treatment.

(G) GABA content in Col-0, HLN1 OE #1 and HLN1 OE #2 plants, with or without drought treatment. (H) Hydrogen peroxide (H_2_O_2_) content in Col-0, HLN1 OE #1 and HLN1 OE #2 plants, with or without drought treatment. (I) Malondialdehyde (MDA) content in Col-0, HLN1 OE #1 and HLN1 OE #2 plants, with or without drought treatment. Data in (B-C) and (F-I) represent means ± standard deviations (SD) from 3 biological replicates. Double asterisks (**) indicate a *p*-value < 0.01, and single asterisk (*) a *p*-value < 0.05 by Student’s *t*-test.


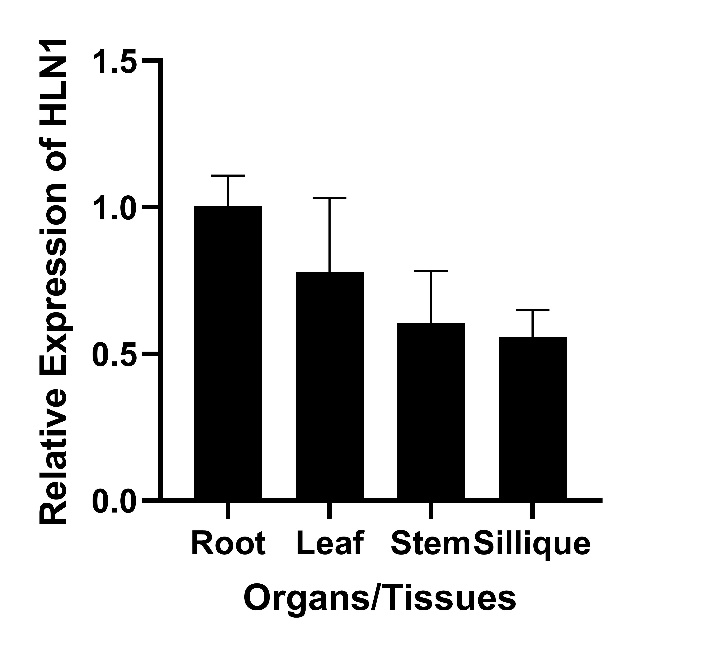


**Supplemental Figure 4**. Relative expression of *HLN1* in different tissues.

Relative expression levels of HLN1 in roots, leaves, stems and siliques of the wild type Arabidopsis (Col-0) plants were determined by qRT-PCR analysis. *ACTIN2* was used as an internal control. Data represent means ± standard deviations (SD) from three biological replicates.


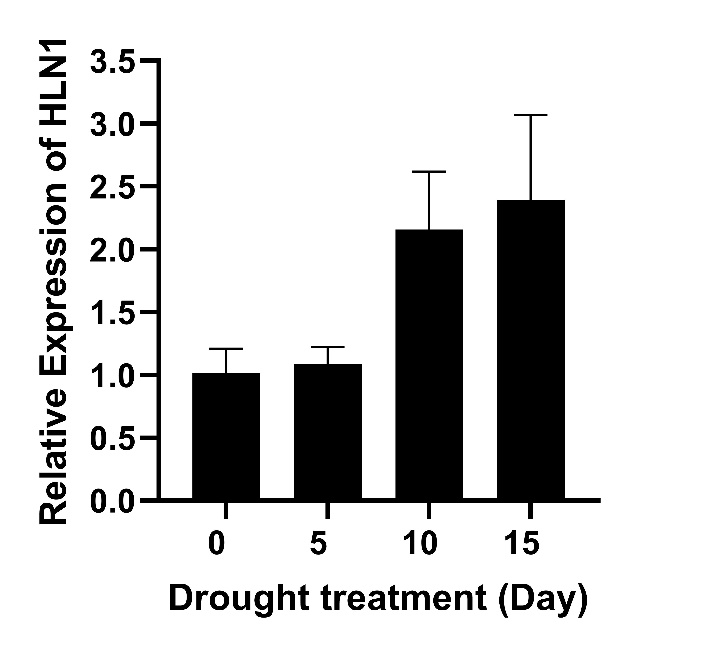


**Supplemental Figure 5**. Expression of *HLN1* was induced by drought stress.

Expression levels of *HLN1* in wild type Arabidopsis (Col-0) plants subjected to 0, 5, 10, 15 days of water-withholding treatment, as determined by qRT-PCR analysis. *ACTIN2* was used as an internal control. Data represent means and standard deviations from three biological replicates.


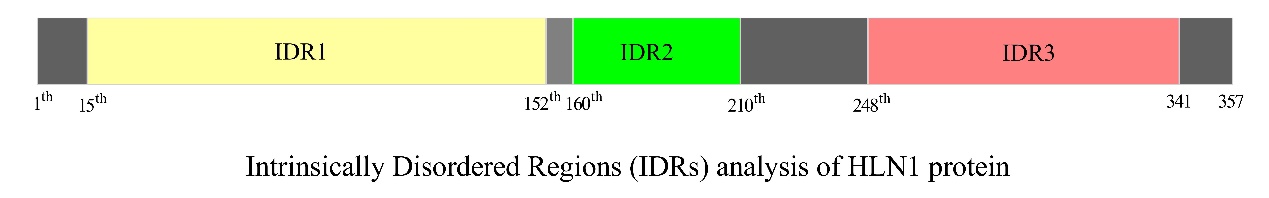


**Supplemental Figure 6.** Intrinsically disordered region (IDR) domain analysis of HLN1.

Schematic presentation of intrinsically disordered regions (IDRs) in the HLN1 protein predicted via PONDR analysis. Numbers correspond to amino acid positions in the protein.


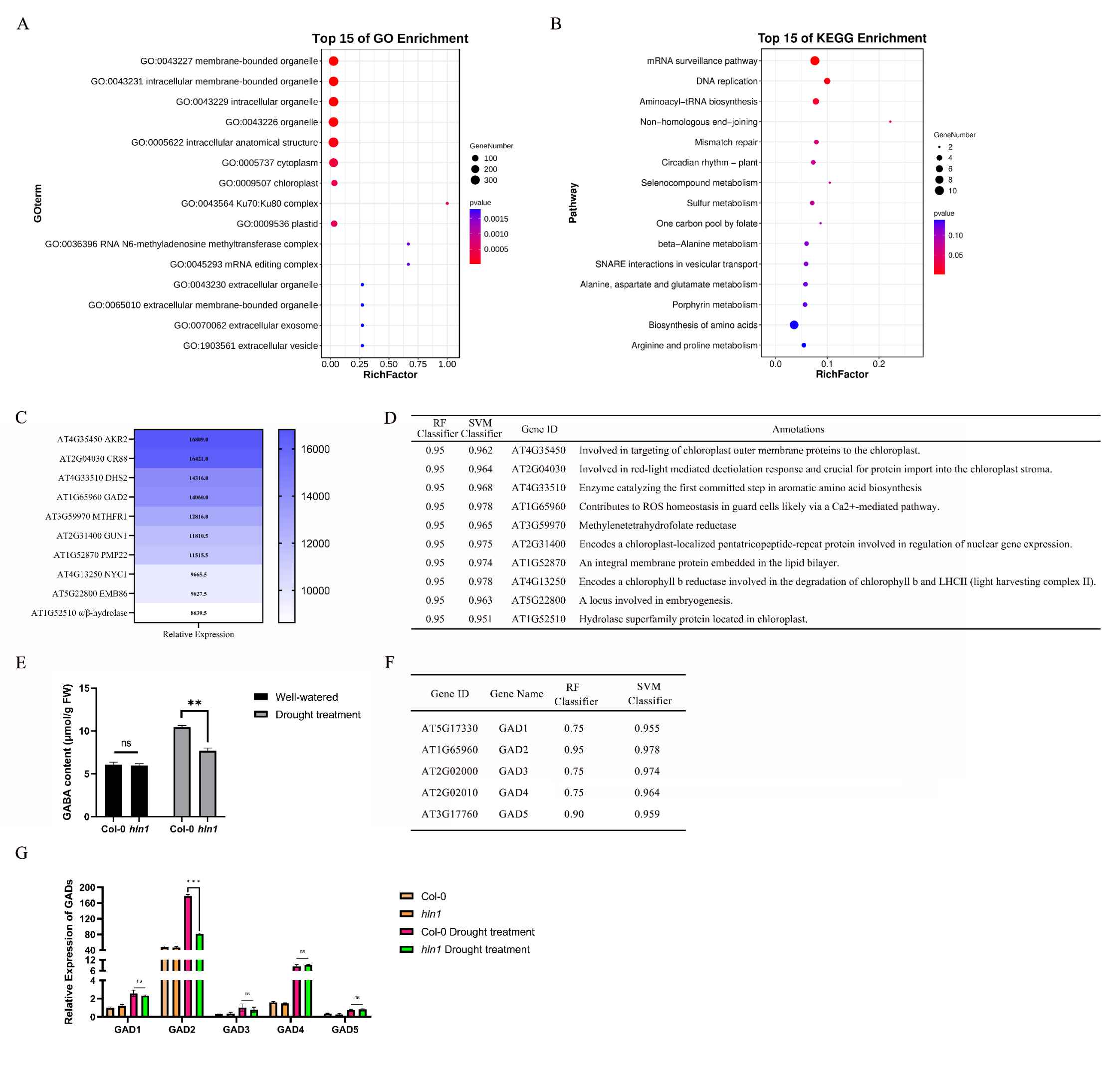


**Supplemental Figure 7.** In-silico analysis of candidate targets of HLN1.

1. Gene Ontology (GO) enrichment analysis of candidate targets of HLN1.

(B) Kyoto Encyclopedia of Genes and Genomes (KEGG) enrichment analysis of candidate targets of HLN1. (C) Heap map analysis of the TOP 10 candidate targets of HLN1 based on leaf transcriptomic data.

(D) The Random Forest (RF) classifier, Support Vector Machine (SVM) classifier, gene ID and annotation of the TOP 10 candidate targets of HLN1.

(E) GABA content in the Col-0 and *hln1* mutant under control and drought stress conditions. Data are means ± SD (*n* = 3). The asterisks (**) represent *p*-value < 0.01 by Student’s *t*-test.

(F) The gene ID, Random Forest (RF) classifier and Support Vector Machine (SVM) classifier of the glutamate decarboxylase (GAD) family members with HLN1.

(G) Relative expression of the glutamate decarboxylase (GAD) family members. Leaves from Col-0 and *hln1* mutant, with or without a 10-day water-withheld treatment, were collected for total RNA extraction and subsequent qRT-PCR analysis. *ACTIN2* was used as an internal control. Data are means ± SD (*n* = 3). The asterisks (***) represent *p*-value < 0.001 by Student’s *t*-test.


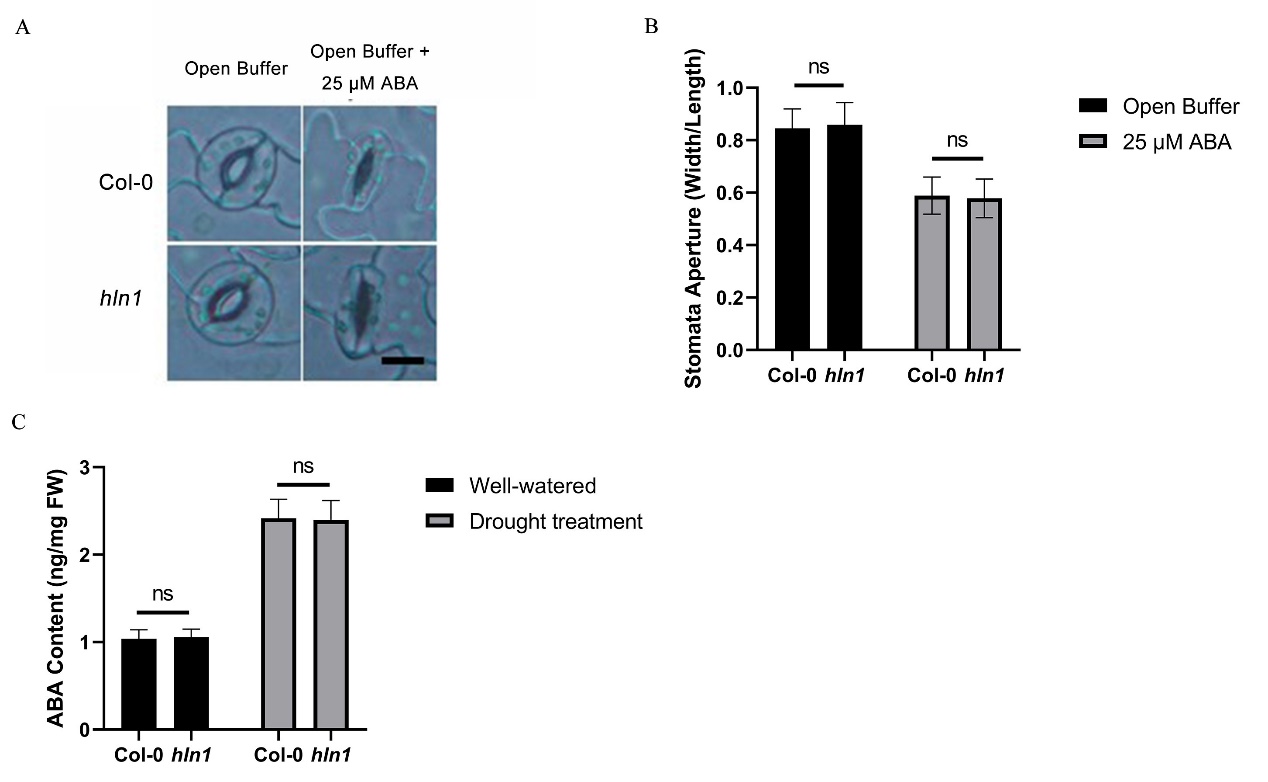


**Supplemental Figure 8. Stomatal response to ABA and ABA content of the wild type and *hln1* mutant under drought stress.**

(A) Stomatal morphology in leaves of 3-week-old Col-0 and *hln1* mutant, with or without 25 μM ABA treatment. The leaves were excised and immersed in Open Buffer for 2 h and transferred to the same buffer supplemented with 25 μM ABA for another 2 h. The epidermal peels were taken for stomatal aperture measurement. Scale bar = 10 μm.

(B) Stomatal aperture (width to length ratio) were calculated by ImageJ (mean ± SD, *n* > 100). The *p*-value was calculated using Student’s *t*-test.

(C) Abscisic acid (ABA) content in Col-0 and *hln1* mutants under control and drought stress conditions (mean ± SD, *n* = 3). *ns*, not significant (*p*-value > 0.05 by Student’s *t*-test).


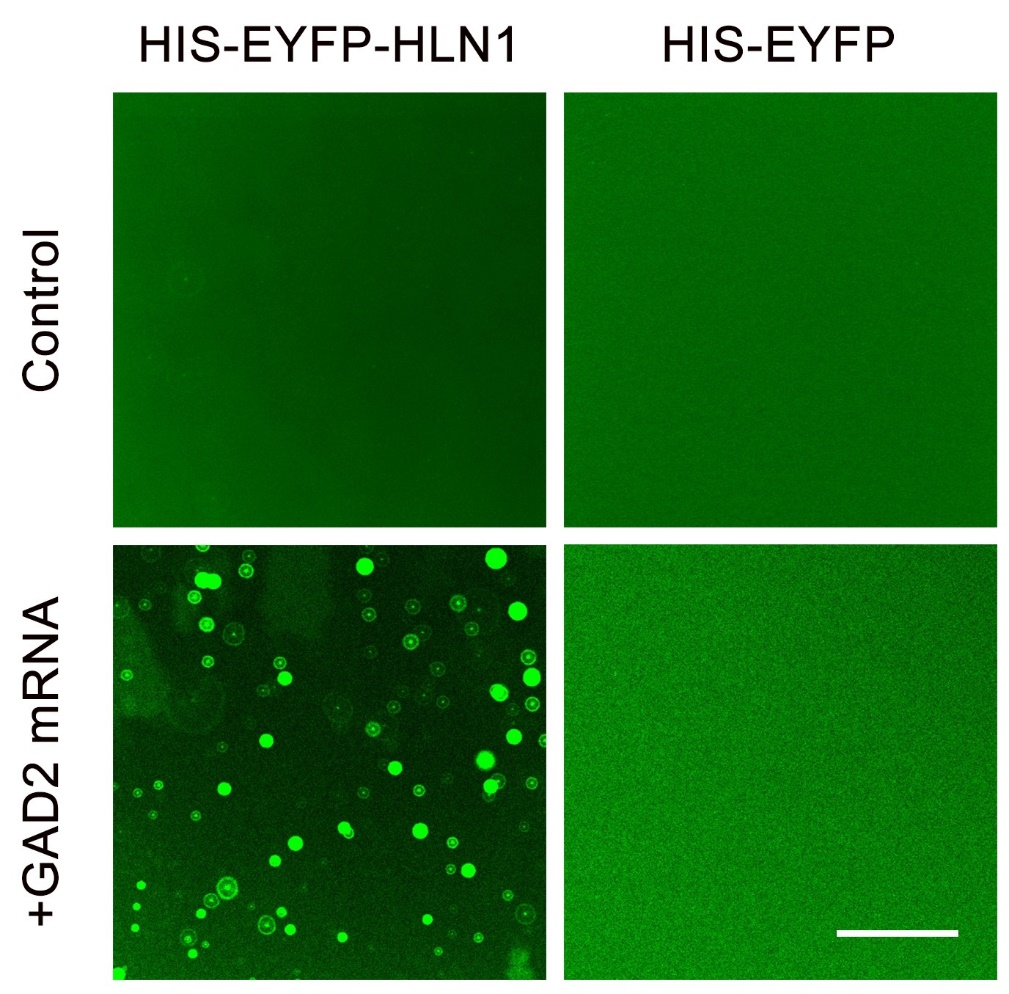


**Supplemental Figure 9.** HLN1 condensates with *GAD2* mRNA in vitro.

Purified HIS-EYFP-HLN1 and HIS-EYFP proteins were incubated with *GAD2* mRNA for 15 min prior to confocal imaging. HIS-EYFP was used as a control. Scale bar = 50 μm.


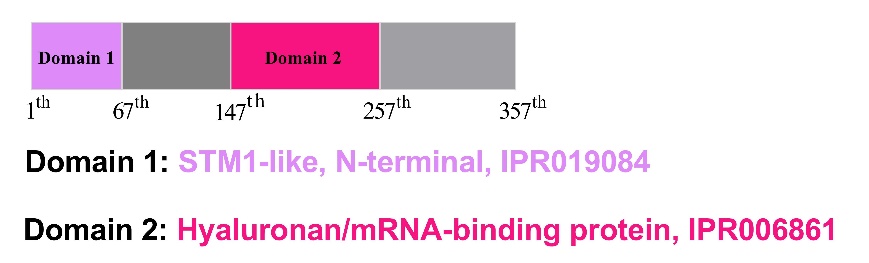


**Supplemental Figure 10.** Functional domain analysis of HLN1.

Schematic presentation of the functional domains in the HLN1 protein predicted via InterPro analysis. Numbers correspond to amino acid positions in the protein.

**Supplemental TABLES**

**Supplemental Table 1.** Primers used in genotyping and qRT-PCR analyses.

| Primer name | Sequence (5' - 3') |
| --- | --- |
| *hln1*-LP | GATATCCCTCCGTAAGCATCC |
| *hln1*-RP | CACTCTTTAATGCCTTCGACG |
| LBb1.3 | ATTTTGCCGATTTCGGAAC |
| ACT2-qF | AAGTCTTGTTCCAGCCCTCG |
| ACT2-qR | TCTGCTGGAATGTGCTGAGG |
| HLN1-qF | TGCTGAGAAGCAAGGAGGTG |
| HLN1-qR | GCTCAACGACTTTTTGGCCT |
| UBQ5-qF | GGTGCTAAGAAGAGGAAGAA |
| UBQ5-qR | CTCCTTCTTTCTGGTAAACGT |
| GAD1 qPCR F | ATGAGCTTCCTTCGAGAGTGAT |
| GAD1 qPCR B | ACTTCTTCCAGCCAGTGATGAT |
| GAD2 qPCR F | TGTGTTCTTCTTCCTCCTCAGA |
| GAD2 qPCR B | TGTAGTGCGAACATAGCGAGAT |
| GAD3 qPCR F | TGCACATTTTTCCCTTTACTTTT |
| GAD3 qPCR B | TCGAATCGTGAGATAGAGTTGC |
| GAD4 qPCR F | TCTTGTTCATTTCAAACCCAAA |
| GAD4 qPCR B | TTTCGAATCGTGGAAGAGAGTT |
| GAD5 qPCR F | AATGGTACTCGCAACCAACTCT |
| GAD5 qPCR B | CATTGATCACTTGATAAGCAGCA |

**Supplemental Table 2.** Primers used to construct plasmids.

| Primer name | Sequence (5' - 3') |
| --- | --- |
| pHLN11-HLN1-no stop-F | ggggacaagtttgtacaaaaaagcaggcttcAGGAGGAGGAGGAGAGAAC |
| pHLN11-HLN1-no stop-F | ggggaccactttgtacaagaaagctgggtgGCCCAACGAAGGGAACTGAGC |
| pHLN11-F | ggggacaagtttgtacaaaaaagcaggcttcAGGAGGAGGAGGAGAGAAC |
| pHLN11-R | ggggaccactttgtacaagaaagctgggtgCACCAGTCTGGTAAGATCGAGT |
| HLN1-with stop-F | ggggacaagtttgtacaaaaaagcaggcttcATGGCGTCTTTGAACCCTTTC |
| HLN1-with stop-R | ggggaccactttgtacaagaaagctgggtgCTAGCCCAACGAAGGGAACTGAGC |
| HLN1-no stop-F | ggggacaagtttgtacaaaaaagcaggcttcATGGCGTCTTTGAACCCTTTC |
| HLN1-no stop-R | ggggaccactttgtacaagaaagctgggtgGCCCAACGAAGGGAACTGAGC |
| EYFP-HLN1-F | atgggtcgcggatccgaattcATGGTGAGCAAGGGCGAGG |
| EYFP-HLN1-R | ctcgagtgcggccgcaagcttGCCCAACGAAGGGAACTGA |
| MBP-HLN1-F | ggcggccgcgatatcgtcgacATGGCGTCTTTGAACCCTTTC |
| MBP-HLN1-R | agggaattcggatccgtcgacTTACTAGCCCAACGAAGGGAAC |
